# Impoverishment impact of out-of-pocket payments for healthcare in rural Bangladesh: Do the regions facing different climate change risks matter?

**DOI:** 10.1371/journal.pone.0252706

**Published:** 2021-06-04

**Authors:** Afroza Begum, Syed Abdul Hamid

**Affiliations:** 1 Department of Statistics, University of Chittagong, Chittagong, Bangladesh; 2 Institute of Health Economics, University of Dhaka, Dhaka, Bangladesh; University of West London, UNITED KINGDOM

## Abstract

**Introduction:**

Out-of-pocket (OOP) payments for healthcare severely affect the current consumption, future health and earnings capacity of poor/underprivileged households and hence it is crucial for priority setting. This study assesses the variation in overall as well as disease-specific impoverishment impact of OOP payments between the regions experiencing different climate change risks, defined as high disaster-prone (HDP) areas and low-disaster-prone (LDP) areas, in Bangladesh.

**Materials and methods:**

This paper estimated three poverty measures, such as poverty headcount, poverty intensity and normalized poverty gap for all ailments, catastrophic events, diseases types (communicable, non-communicable (NCDs), and accident and injury), illness conditions (acute and chronic) and hospitalization using 3,791 randomly selected rural households (1,203 from HDP and 2,588 from LDP areas) across the regions. Cost of basic need approach was used for estimating poverty line expenditure.

**Results:**

About 13 percent households annually fall into poverty due to OOP outlays for healthcare. Despite having significantly (*p-value≤0*.*01)* less OOP payments (HDP areas: BDT 5,117; LDP areas: BDT5,811) the impoverishment impact of OOP payments for healthcare in HDP areas (16.5%) has substantially higher than LDP areas (11.3%).

Population in HDP areas, especially *char* (river island; 19.55 percent) and *haor* (water submerged; 16.80 percent) are more susceptible to any level of OOP payments due to low level of earnings. Catastrophic healthcare expenditure (61.79%) and NCDs (14.29 percent) are exacerbating the poverty level in Bangladesh. Both absolute and relative average poverty gap are more widen in HDP than LDP areas due to catastrophic OOP outlays for healthcare.

**Conclusion:**

The impoverishment effect due to OOP payments for healthcare in both HDP and LDP areas are high, especially for NCDs and catastrophic healthcare expenditure. However, the situation is bit worse in HDP areas. Preventing the escalation of NCDs as well as catastrophic expenditure and hence reducing the level of impoverishment thereof call for restricting tobacco use, increasing physical activity, encouraging to intake healthy diets, ensuring food safety, controlling air pollution, and improving mental health. Moreover, government should give more emphasis, especially in the HDP areas, on making community clinics more functional through providing screening equipment and training to the Community Health Care Providers for early detection of NCDs, and ensuring availability of medicine all the time. Note that other than community clinics, there is little option for providing healthcare in HDP areas due to poor functionality of public facilities as well as lack of private facilities in HDP areas.

## Introduction

In low- and middle-income countries, Out-of-pocket (OOP) payments are the leading source of healthcare financing [1,2]. OOP payments accounts for about 74 percent of total healthcare expenditure in Bangladesh [3] while the global average is only 32%. [4]. Bangladesh has the highest share of OOP payments in total healthcare expenditure among South-East Asia region, followed by India (62%) [5]. OOP payments for health care severely affect consumption of the poor households during the periods of major illness or forces the poor to forego treatment, which raises the chance of long-term deterioration in health and earnings capacity [6–8]. Out-of-pocket payment therefore is claimed to be a major cause of poverty [9] or aggravating poverty [10–12]. The choice of a coping strategy also has implications on the poverty outcome [13]. Evidence shows that rural households in Bangladesh bear a massive burden of OOP outlays for healthcare, which pushes annually 3–6 percent of the households into poverty [12,14]. Quite large OOP burden is also evident in India [11,12,15,16] and some other Asian countries [12].

A large number of studies in the developing counties have documented the impoverishment impact of OOP payments [10–12,13–31]. Some of these studies depicted the variation of OOP burden among some countries of Asia [12] and East Europe [32] and some studies depicted the regional inequities in public health care financing [11,33–35].

A number of studies also concentrated in assessing the impoverishment impact of OOP payments for healthcare in Bangladesh context [14,24,26,36–38]. Hamid et al (2014) mainly focused on disease-specific impoverishment in the rural areas [14]. Another study measured the non-communicable diseases related impoverishment impact using the Household Income and Expenditure Survey (HIES) 2010 data [26]. Using the same national survey data, Khan *et*. *al*. (2017), and Molla and Chi (2020) explored the overall national impoverishment impact of OOP payments providing more emphasis on catastrophic healthcare expenditures [24,37]. Based on HIES 2016 data, Sayem *et al*., 2021 made rural-urban as well as regional comparisons of impoverishment impact of OOP outlays for healthcare at the divisional level and found geographical location as one of the important factors of impoverishment [38]. Moreover, several studies examined the level of OOP payments and/or catastrophic healthcare expenditures [39–42]. It seems that the existing literature in Bangladesh context covers rural-urban and/or divisional comparisons rather than ecological diversity of the country for assessing the impoverishment impact of OOP payments for healthcare.

Bangladesh, despite being a very small country, has a vast ecological diversity, e.g., plain land, river bank and/or river island (*char*), water submerged (*haor*) and coastal areas. The latter three areas experience high climate change risks where the incidence of natural calamities (e.g., flood, cyclones, droughts, river bank erosion, salinity etc.) is higher. Note that OOP outlays for healthcare would plausibly vary across the regions experiencing different climate change risks, a major threat to the human health [43,44]. Accordingly, one would expect, that the impoverishment impact of OOP outlays may vary across the regions facing such risks. However, there is hardly any evidence in this regard despite its enormous importance *for priority setting* in any informed policy discussion. Such evidence also does not exist in the international context. Indeed, the lack of this evidence, particularly in Bangladesh context, motives the present paper. The paper, thus, aims at exploring the variation of poverty impact of OOP spending for healthcare across the ecological diversified areas of Bangladesh which experiences different climate change risks. More specifically, this paper explores the variation of OOP payments related burden for healthcare between the regions facing high climate change risks and low-climate change risks. This can be defined respectively as high disaster-prone (HDP) areas and the low-disaster-prone (LDP) areas. The study also examines the disease-specific impoverishment impact of OOP payments between the two aforementioned areas.

## Materials and methods

### Study design and setting

This study used cross-sectional data from the second-round survey of a longitudinal research project conducted during May-June 2011. The survey used multi-stage stratified sampling design under the framework of Integrated Multipurpose Sample design.

In this survey, we included five *unions*, the smallest administrative unit in Bangladesh, from the first-round survey located in three districts (Mymensingh, Tangail and Brahmanbaria) spread over two divisions of the country which comprises a sample of 2000 households. Capturing the geographical diversity along with making it nationally representative is the main reason for not including the full sample of the first-round survey conducted in 2009 over two divisions (details are illustrated in [45]). Additionally, 2200 households were included in second-round survey from 5 new randomly selected districts (Chapainawabganj, Chuadanga, Patuakhali, Sunamganj and Gaibandha) in order to capture all the seven divisions of the country and also keeping in view the geographical diversity (c*har*, *haor*, costal and boarder areas). One *upazila* from each district and two *unions* for each *upazila* were selected randomly which yielded a total of ten *unions* from the 5 newly selected districts. To match the sampling process of the first-round survey, the newly included *unions* were divided into ten strata (five with ‘in-patient’ care facility and five without in-patient facility). A sampling frame was formed by listing all the villages in each *union*. A sample of 7 villages was randomly selected from each *union* with in-patient facility and 5 villages from each *union* without such facility.

The sample size for each stratum was estimated using the following formula

n=t(α,N−1)2P(1−P)d21+1N(t(α,N−1)2P(1−P)d2−1)*Designeffect

with the population size *N* collected based on a sampling frame formed in April 2011, level of significance *α* = 0.05 and margin of error *d* = 0.5 and design effect = 1.5. It is noted that, this survey was a multipurpose survey where the main outcome was ‘the proportion of individuals seek healthcare for general illnesses. Thus, we select this outcome as the parameter *P* where its value is considered as 0.3 based on the findings of first round survey.

Thus, using equal allocation, the research team randomly selected 440 households from each 5 new selected districts yielding 2,200 (440*5) additional households in total. The total sample size in the second round survey thus stands at 4,200 (2,000 + 2,200) households from 120 villages of 8 districts over 7 divisions.

We also conducted a village survey which covered details of physical, educational and health infrastructures, literacy rate, macro shocks occurred (floods, droughts, cyclones, river erosions, pest attack and so on) and the type of insurance products available locally.

### Data collection

We used a semi-structure questionnaire which was developed in native language (Bengali). After internal review, the questionnaire was sent to a group of experts for external review. Incorporating the experts’ feedbacks, the revised questionnaire was made ready for the training of interviewers. After a thorough checking of inconsistencies and language suitability during training sessions, the questionnaire was geared up for testing. Incorporating the feedback received from the piloting process, the questionnaire was then finalized before being administered to the subjects. The survey was conducted via interviewers of 10 groups, each consisting of a field supervisor and 4 field investigators. The core research team visited all the survey areas to ensure the quality of the data collection process. In addition, a research assistant made unannounced field visits and verified the questionnaires from time to time. The data were finalized for the analysis after necessary editing and coding, consistency checking by the core research team.

A series of questions regarding OOP payments were posed to the respondents for each episode of illness within the household over the 12 months recall period. The recall period for health care utilization needs to be fixed to satisfy the dual objectives: minimizing the recall bias and maximizing the sample of target subjects [46]. In the literature of impoverishment impact of OOP payments, some used 12 months recall period for both inpatient and outpatient cases [19] while some preferred 1 month recall for outpatient and 12 months for hospitalization cases [20,23]. In a study on 11 Asian countries van Doorslaer et al. (2006) used different recall periods (varying from 1 month to 12 months) for different countries while for Bangladesh they used 1 month for both cases [12]. Similarly, Yardim et al. (2010) used 1 month for both inpatient and outpatient care [22]. Information on morbidity, health care seeking pattern, demographic condition, occupation, education, income, expenditure, assets, borrowing, etc. from the households were also collected. Household heads were the main respondents.

The study received ethical approval from the Institutional Review Board of Institute of Health Economics, University of Dhaka, Bangladesh. Verbal consent of the participants was solicited by informing about the procedures and risks involved in the study.

### Methods of data analysis

#### Variables

In measuring out-of-pocket payments, we mainly considered the payments for direct medical inputs used by the sick. More precisely, out-of-pocket payments was constructed by adding the expenses that a household incurred for consultations, drugs, diagnostic tests, surgical operations, and bed charge for each episode of illness for the 12 months preceding the survey. This type of out-of-pocket payments may be termed as direct out-of-pocket payments. Total out-of-pocket payments may be constructed by adding the payments for transportation and other (food, lodging, accommodation and unofficial fees) with direct out-of-pocket payments. Out-of-pocket payments for communicable diseases (CDs), non-communicable diseases (NCDs), accidents and injuries (A&I) chronic illnesses, acute illnesses, hospitalization and catastrophic illnesses were constructed by adding the relevant expenses incurred for each episode of illness in each category. We used both WHO fact sheets and CMS (Council for Medical Schemes) guidelines to define chronic diseases.

We used both food and non-food expenditure as a proxy for household income. For measuring food expenditure, we considered the expenditure on all the food bundles consumed by the household for the week preceding the survey. We considered expenditure for non-food expenditure against the following items: clothing, toiletries, cookware, blankets, furniture, lamp, torch light, candle, match, kerosene, electricity, transportation, fuel, maintenance and repairing of household materials, taxes, donation and tolls, recreation, tobacco, tuition fees, stationeries, mobile and land telephone bills, festivals and traditional ceremonies, electronic equipment and health expenses (both direct and indirect).

#### Poverty line

We used Cost of basic need (CBN) approach developed in a prior study [47]. This method was also used for estimating the poverty line expenditure in all the reports of Bangladesh Household Income and Expenditure Survey (HIES) 2005, 2010 and 2016 [48–50]. Following the reports of HIES, we estimated the poverty line expenditure by CBN method. The estimated per day per capita poverty line expenditure was BDT 62, BDT 57, BDT 62, BDT 47 and BDT 48 for LDP districts (Tangail, Mymensingh, Brahmanbaria, Nawabganj, and Chuadanga) respectively. It was BDT 45, BDT 49 and BDT 57 respectively for the three HDP areas (*char*, *haor* and coastal). However, we used the average poverty line expenditure for determining the incidence and depth of poverty. The average poverty line expenditure was BDT 50.3 per day per capita and BDT 55.2 per day per capita in HDP and LDP respectively while overall average poverty line expenditure was BDT 52.75 per day per capita.

#### Measurement of impoverishment impact of out-of-pocket payments for health care

We estimated the impoverishment impact of OOP payments for respective category of illness by comparing the difference between the average level of head count poverty or poverty gap (intensity of poverty or poverty deepening) before health care payments and after payments following earlier studies [4–6]. Headcount poverty measures the percentage of individuals or households living below the poverty line, while poverty gap measures poverty deepening or intensity of poverty (the amount by which the poor households fall short of attaining the poverty line). Pre-payment headcount poverty $\left(H_{Pov}^{pre}\right)$ was calculated by comparing per capita household expenditure (*including OOP payments for health care*) with a poverty line estimated by the authors. It is noted that we included health expenses (both direct and indirect) for pre-payment poverty measurement and excluded direct health expenses for the post-payment measurement. Similarly, the post-payment headcount poverty $\left(H_{Pov}^{post}\right)$ was measured by comparing per capita household expenditure (*excluding direct out-of-pocket payments for health care*) with the poverty line. This is to note that the pre-payment health care financing (e.g., insurance) mechanism usually does not cover expenses like transportation cost, and cost for food, lodging, accommodation and unofficial fees. In order to link policy discussion with the insurance mechanism we did not include this part of health care expenses. Thus, we meant ‘direct out-of-pocket payments’ as OOP payments in the remaining part of the text.

Assume *z_i_* to be the per capita expenditure (*including OOP payments for health care*) for an individual, *p_L_* is the poverty line and *n* is the number of individuals. Pre-payment and post-payment headcount poverty measures can be expressed respectively as

$$H_{Pov}^{pre}=1/n\sum_{i=1}^{n}z_{i}\leq p_{L}$$ (1)

$$H_{Pov}^{post}=1/n\sum_{i=1}^{n}(z_{i}-OOP)\leq p_{L}$$ (2)

Similarly, the pre-payment and post-payment poverty gap can be defined respectively as

$$G^{pre}=1/n\sum_{i=1}^{n}\rho_{i}(p_{L}-z_{i});$$ (3)

$$G^{post}=1/n\sum_{i=1}^{n}\rho_{i}\{p_{L}-(z_{i}-OOP)\};$$ (4)

where *ρ_i_* = 1 if *z_i_*≤*P_L_* and *ρ_i_* = 0 if *z*_*i*_> *P*_*L*_.

The headcount poverty is higher in Eq (2) compared to Eq (1) if OOP outlays are positive. Similarly, the poverty gap is higher in Eq (4) compared to Eq (3). Thus, the difference between Eq (2) and Eq (1) depicts headcount impoverishment impact of OOP payments. Similarly, the difference between Eq (4) and Eq (3) illustrates the intensity of poverty on account of OOP payments. More precisely, headcount and poverty gap impoverishment impact of OOP payments can be expressed respectively as ${(H}_{pov}^{post}-H_{pov}^{pre})$ and (*G^post^−G^pre^*). It is useful to use normalized poverty gap (the size of poverty gap in relation to poverty line, (*G^post^−G^pre^*)/*P_L_*, for a comparative analysis.

## Results

### Characteristics of the sample areas

Historically, riverbank and/or river island (*Char*), water submerged (*Haor*) and coastal areas are considered as disaster-prone areas where climate change risk (like flood, cyclones, droughts, riverbank erosion, salinity etc.) is more prevalent. Note that Gaibandha, Sunamganj and Patuakhali districts respectively belong to *char*, *haor and* coastal areas. Thus, we classified the survey areas, based on climate change risk, primarily into two categories: high-disaster-prone areas (Gaibandha, Sunamganj and Patuakhali) and low-disaster-prone areas (Tangail, Mymensingh, Brahmanbaria, Nawabganj and Chuadanga).

A justification of this claim has been made by the village survey which was conducted to capture the occurrences of various disasters including flood and cyclone during the last five years preceding the survey.

As seen in Table 1 that 89, 42, 32 and 21 percent of the villages in HDP areas, as defined earlier, affected by flood (the core disaster in Bangladesh), cyclone, drought, and riverbank erosion respectively during five years preceding the survey. In the LDP areas, the occurrences of these disasters, especially the flood, are significantly (*p-value* ≤ 0.01) lower compared to HDP areas. This clearly implies that districts included in the HDP and LDP areas are justified.

**Table 1:** Percentage of villages affected by various disasters during the five years preceding the survey.

Nature of disaster	Total % (n = 100)	HDP Areas				LDP Areas (62)
		*Char* (12)	*Haor* (14)	Coastal (12)	Total (38)	
Flood	37 (37)	66.67 (8)	100.00 (14)	100.00 (12)	89.47 (34)	4.84 (3)
Cyclone	38 (38)	33.33 (4)	0.00 (0)	100.00 (12)	42.11 (16)	35.48 (22)
Drought	33 (33)	33.33 (4)	0.00 (0)	66.67 (8)	31.58 (12)	33.87 (21)
Riverbank erosion	9 (9)	16.67 (2)	0.00 (0)	50.00 (6)	21.05 (8)	1.61 (1)

Note: The information of climate shocks from a number of 100 villages is reported here though the household survey was conducted on 120 villages. The data of 20 villages of low-disaster-prone areas are not available.

### Key characteristics of the sample households

A total of 3,791 households with 18,301 individuals, of which 1,203 households from HDP areas (400,404 and 399 households from *char*, *haor* and coastal areas respectively) and 2,588 from LDP areas were successfully interviewed. Majority (62.8%) of the cases, household heads were the main respondents while spouses were for 29.9 percent of the cases. Most (about 92%) of the households were male-headed. Average education level of the household head was about 3 years and average age was 46 years. These figures are slightly lower in HDP areas compared to LDP areas.

The average household size was 4.49 and the average per capita daily expenditure (both food and non-food) was about BDT78.7. The former is significantly (*p-value ≤*0.05) higher in HDP areas compared to the LDP areas while the latter is significantly (*p-value ≤0*.*01)* lower in HDP areas compared to the LDP areas. There is also quite large variation in per capita daily expenditure among the HDP areas. This is BDT 60.16, 63.03 and 80.5 in *Char*, *Haor*, in coastal areas respectively (results are not shown in Tables).

### Pattern of morbidity and health care seeking

The survey collected the information on 18,301 individuals of which about 49 percent underwent some form of self-reported morbidity over 12 months. Further noted that overwhelming majority (98.6%) of the households experienced some sort of morbidity. There is no significant variation in morbidity between HDP (48%) areas and LDP (49%) areas. However, there is a large variation among the HDP areas (70%, 42% and 37% in *char*, *haor* and coastal areas respectively). About one-third of the sick suffered from ‘general cough and fever’. Other major symptoms were gastrointestinal disorder, diarrhea, weakness, abdominal pain, typhoid and blood pressure, asthma, heart disease etc.

The incidence of CDs, NCDs, A&I, and chronic illness was 51.5%, 45.7%, 2.8% and 16% respectively. No significant differences were found in the incidence of broad types/conditions of illnesses between HDP and LDP areas. However, there are quite large differences in some of these broad types of illnesses among the HDP areas. For example, the incidence of chronic illnesses was about 20 percent in coastal, 15 percent in *char* and 14 percent in *haor* areas. The overwhelming majority (about 99%) of the sick sought some kind of health care. However, most (about 94%) of the care-seekers went for outpatient services irrespective of the regions facing different climate change risks. Almost all the affected households (99.7%) or sampled households (98.3%) sought some health care. Any significant variation was not seen in health care seeking pattern for outpatient and inpatient cases between the HDP areas and LDP areas (results are not shown in Tables).

### Out-of-pocket payments for healthcare

The results show that OOP payments for healthcare as a whole and drug related OOP payments of per *affected* household for all episodes of illnesses over the 12 months preceding the survey was BDT5,590 and BDT3,895 respectively (Table 2). It is worth mentioning that 1 US $ was equivalent to BDT 74 at the time of the survey. OOP payments for healthcare as a whole and OOP costs on account of drugs for all episodes of illnesses over 12 months, when averaged over all sampled households, the figures decline to BDT 5,494 and BDT 3,829, respectively (results are not shown in the table). The cost of drugs thus appears to be the major component of OOP payments for healthcare (about 70%), which is significantly (*p-value ≤0*.*01*) higher in HDP areas (about 74%) compared to LDP areas (about 68%). In terms of annual expenses, OOP payments for healthcare were 4.61 percent of the total household food and non-food expenditure and 8.68 percent of the food expenditure. Although the average OOP outlays for healthcare were significantly (*p-value ≤0*.*01)* lower in HDP areas (BDT 5,117) than LDP areas (BDT5,811), there was no substantial difference in its proportion to total household expenditure. There were not considerable variations in OOP payments for healthcare among the HDP areas (BDT 5,348, BDT5,129 and BDT4, 869 in *char*, *haor* and coastal areas respectively). However, households in the *char* areas were needed to spend above 6 percent of total household expenditure and above 10percent of food expenditure while the corresponding figures were significantly lower (*p-value ≤0*.*01)* in both *haor* and coastal areas. Thus, it appears that *char* areas were more vulnerable in terms of share of OOP expenses of household food and non-food expenditure.

**Table 2:** Out-of-pocket payments per *affected* household by the geographical areas.

Types of geographical areas		Mean OOP payments (in BDT) over12 months	Mean of drug expenses (in BDT) over 12 months	OOP payments as % of total household food and non-food expenditure	OOP payments as % of food expenditure	Drugs expenses as % of OOP payments
HDP areas	*Char*	5,348 (7,954)	4,074 (5,035)	6.30 (398)	10.35 (398)	76.17 (398)
	*Haor*	5,129 (6,532)	3,853 (4,292)	4.14 (403)	6.93 (403)	75.12 (403)
	Coastal	4,869 (7,584)	3,493 (5,066)	4.08 (387)	7.06 (387)	71.74 (387)
	Total	5,117 (7,372)	3,809 (4,808)	4.68 (1,188)	7.89 (1,188)	74.44 (1,188)
LDP areas		5,811 (12,569)	3,936 (7,489)	4.58 (2,538)	9.04 (2,538)	67.73 (2,538)
Total		5,590 (11,181)	3,895 (6,750)	4.61 (3,726)	8.68 (3,726)	69.69 (3,726)

Note: 1. Household food and non-food expenditure has been scaled up to 12 months.

2. Figures in parentheses are standard errors in columns 2–3 and number of observations in 4–6.

Table 3 displays the average OOP payments for broad types of illnesses/types of care: CDs, NCDs, I&A, acute, chronic, inpatient and outpatient. It is seen that per episode of NCDs and A&I accounted for significantly (*p-value<0*.*01*) higher OOP payments (BDT 5,938 and BDT5, 871 respectively) than CDs (BDT 1,490). Presumably a good part of the A&I expenses would be for whatever ‘emergency care’ that can be accessed at the time. OOP payments for per episode of *chronic* condition (BDT 6,603) were significantly (*p-value<0*.*01*) higher than *acute* conditions (BDT 3,782); and similar difference was found between inpatient and outpatient care.

**Table 3:** Out-of -pocket payments by types of illnesses, types of care and geographical areas.

Types of geographical areas		Types of diseases			Illness conditions		Types of care	
		CDs	NCDs	I & A	Acute	Chronic	Inpatient	Outpatient
HDP areas	*Char*	1,556 (2,729)	4,828 (6,203)	4,675 (11,933)	3,622 (6,327)	5,668 (6,607)	7,673 (12,736)	4,301 (5,306)
	*Haor*	1,674 (2,982)	4,501 (5,606)	7,247 (12,053)	4,164 (6,115)	4,250 (4,021)	9,307 (11,253)	3,932 (4,295)
	Coastal	1,850 (4,256)	5,194 (8,069)	6,472 (6,180)	3,550 (6,148)	5,838 (8,383)	10,425 (11,004)	3,848 (5,760)
	Total	1,677 (3,287)	4,819 (6,620)	5,748 (10,826)	3,786 (6,200)	5,273 (6,606)	8,997 (11,738)	4,030 (5,143)
LDP areas		1,405 (3,935)	6,491 (13,500)	5,954 (9,756)	3,781 (9,284)	7,204 (12,919)	16,633 (25,507)	4,008 (7,116)
Total		1,490 (3,747)	5,938 (11,707)	5,871 (10,175)	3,782 (8,427)	6,603 (11,370)	14,007 (22,061)	4,015 (6,549)

Note: Figures in round parentheses are standard errors.

Average OOP payments for healthcare for an episode of NCDs, chronic condition and inpatient care were significantly(*p-value<0*.*01*) lower in HDP areas compared to LDP areas while the differences were not prominent among other types of illnesses. There were quite large variations in average OOP payments for healthcare among the HDP areas, especially for inpatient care. The average OOP outlays for healthcare for an episode of inpatient care was BDT10, 425 in coastal areas while the corresponding figure for *char* and *haor* respectively wasBDT7,673 and BDT9,307.

Table 4 shows the incidence of illness that leads to catastrophic health expenditures. About 16 percent of the sampled households incurred catastrophic healthcare expenditure at the 10 percent threshold level over 12 months recall period. This is to note that NCDs is the main contributing factor for catastrophic healthcare expenditure because among these households, about 81percent had any NCDs cases. Moreover, healthcare related average OOP payments for the households incurred catastrophic health expenses were BDT19,520. This was significantly (p-value<0 .01) higher in LDP areas compared to HDP areas. There was, in fact, no difference in the incidence of catastrophic health payments between the sampled and affected households since almost all the households had some kind of illnesses and sought some form of healthcare (Table 4). The incidence of catastrophic healthcare expenditures was quite higher in HDP areas (17.9%) than LDP areas (15.3%). Among the HDP areas about 26 percent of the households in *char* areas incurred catastrophic health care expenditures while the corresponding figure in coastal and *haor* was16.5 percent and 11 percent respectively.

**Table 4:** Incidence of catastrophic health expenses (measured at 10% threshold level) by geographical areas.

Types of geographical areas		Incidence of catastrophic health payments among the affected households (%)	Incidence of catastrophic health payments among the sampled households (%)	Average OOP payments of the catastrophic households
HDP areas	*Char*	26.13 (104)	26.00 (104)	12,676 [12,182) (104)
	*Haor*	11.17 (45)	11.14 (45)	16,760 [10,617] (45)
	Coastal	17.05 (66)	16.54 (66)	15,764 [12,393] (66)
	Total	18.1 (215)	17.87 (215)	14,479 [12,19] (215)
LDP areas		15.64 (397)	15.34 (397)	22,251 [25,45] (397)
Total		16.43 (612)	16.14 (612)	19,520 [21,700] (612)

Note: Figures in round parentheses are number of observations and in the squared parentheses are standard errors.

### Impact on poverty

#### Headcount impoverishment impact of out-of-pocket payments

**Fig 1:**
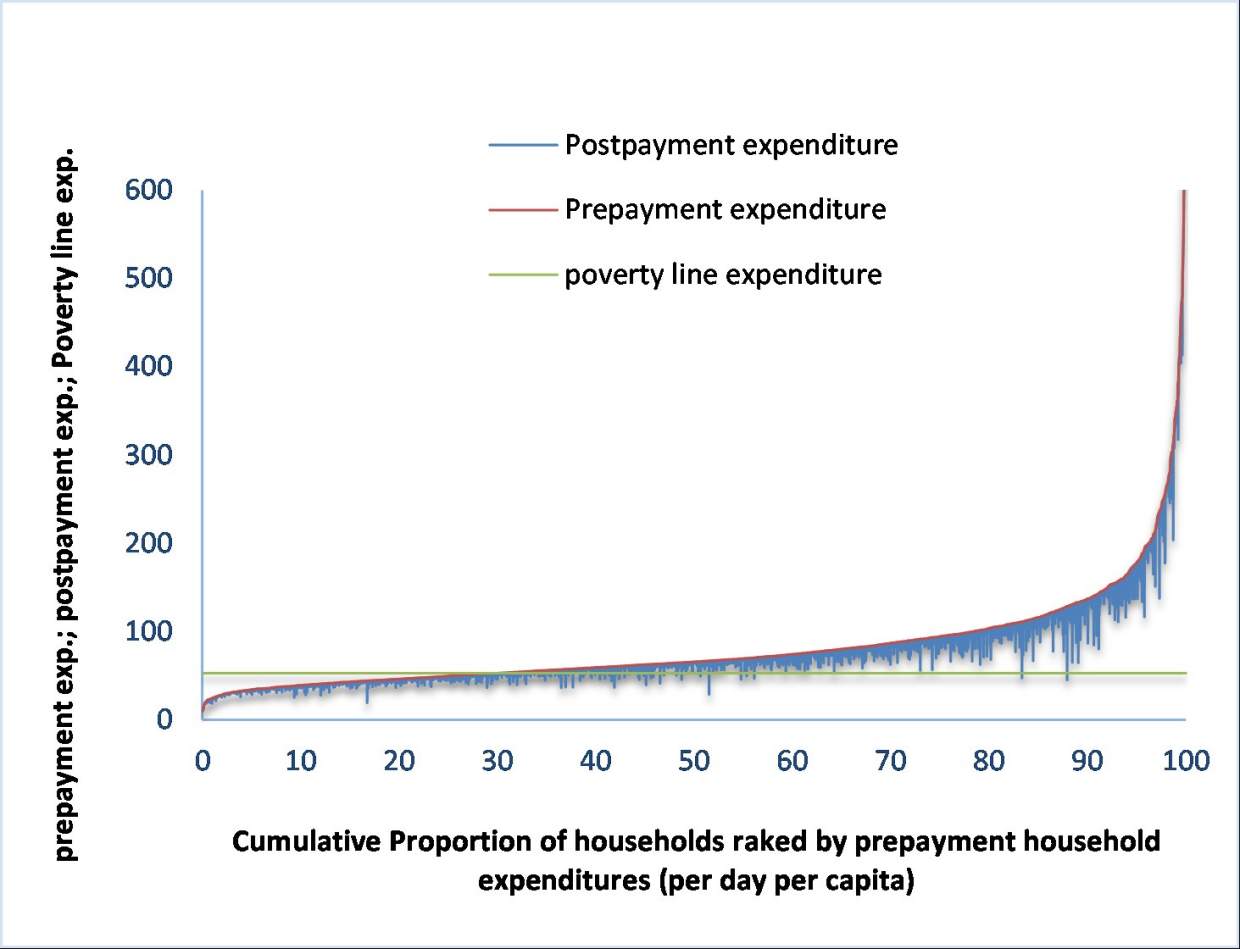
Poverty Pen’s parade graph for all ailments.

Overall pre-payment headcount poverty was 31.07 percent and post-payment (after deduction of expenses for health care from total household expenditure) headcount poverty was 35.14 percent (Table 5, columns 3–4). Thus, 13.1 percent (absolute poverty: 4.07 percentage points) households fell into poverty due to payments for health care, which was statistically significant at five percent level. The impoverishment impact of payments for health care can be observed by plotting pre-payments as well as post-payments household expenditure against cumulative proportion of the households ranked by pre-payment consumption expenditure (per day per capita) in Pen’s parade graph. Pen’s parade graph reveals the extent to which households are pulled below the poverty line and further pushed below and made them poorer who are already below the poverty line. Fig 1 shows the Pen’s parade graph for the pre-payments and post-payments household expenditure. The point at which the pre-payment parade intersects with the poverty line indicates the pre-payment headcount poverty. The ‘paint drops’ from the prepayment curve portray that payments for health care drag the household expenditure down the pre-payment level. It is clear from the graph that health care expenses pushed many non-poor households (including higher income households) into poverty.

**Table 5:** Impact of OOP payments on poverty for all ailments and catastrophic events by geographical areas.

Particulars	Types of geographical areas		Poverty Headcount or Poverty Incidence (in %)				Poverty Intensity or Average Poverty Gap (in BDT)			Poverty impact (Normalized poverty gap (in %))
			Pre-payment	Post- payment	Poverty headcount impact in percentage points (Absolute Poverty)	Poverty headcount impact in percent (Relative Poverty)	Pre-payment	Post- payment	Poverty impact (intensity)^¶^	
							Mean [SD] (n)	Mean [SD] (n)		
All Ailments	HDP areas	*Char*	33.25 (133)	39.75 (159)	6.5	19.55	13.59 [6.06] (133)	14.33 [6.41] (159)	0.74 (5.45%)	1.64
		*Haor*	35.40 (143)	41.34 (167)	5.94	16.78	10.49 [5.93] (143)	10.75 [6.07] (167)	0.26 (2.48%)	0.53
		Coastal	32.58 (130)	36.84 (147)	4.26	13.08	16.1 [8.34] (130)	16.47 [8.32] (147)	0.37 (2.30%)	0.65
		Total	33.75 (406)	39.32 (473)	5.57*	16.50	13.30 [7.20] (406)	13.73 [7.33] (473)	0.43 (3.23%)	0.85
	LDP areas		29.83 (772)	33.19 (859)	3.36	11.26	15.82 [9.29] (772)	16.33 [9.43] (859)	1.11** (7.02%)	2.01
	Total		31.07 (1,178)	35.14 (1,332)	4.07**	13.10	14.95 [8.71] (1,178)	15.41 [8.83] (1,332)	0.46 (3.08%)	0.87
Catastrophic events	HDP areas	*Char*	29.41 (30)	43.14 (44)	13.73	46.68	12.57 [5.85] (30)	14.95 [6.70] (44)	2.38 (18.93%)	5.29
		*Haor*	24.44 (11)	37.78 (17)	13.34	54.58	8.68 [7.75] (11)	11.63 [6.66] (17)	2.95 (33.99%)	6.02
		Coastal	24.24 (16)	39.39 (26)	15.15	62.50	15.23 [8.97] (16)	16.29 [8.60] (26)	1 (6.57%)	1.75
		Total	26.51 (57)	40.47 (87)	13.96*	52.66	12.57 [7.41] (57)	14.70 [7.41] (87)	2.13* (16.95%)	4.23
	LDP areas		18.64 (74)	31.49 (125)	12.85**	68.94	15.87 [9.91] (74)	17.38 [10.70] (125)	1.51 (9.51%)	2.74
	Total		21.41 (131)	34.64 (212)	13.23***	61.79	14.43 [9.03] (131)	16.28 [9.56] (212)	1.85* (12.82%)	3.51

Note

^¶^ The figure in parentheses refers the poverty impact in percentage.

***, **, and * indicates significance at 1%, 5%, and 10% respectively.

**Fig 2:**
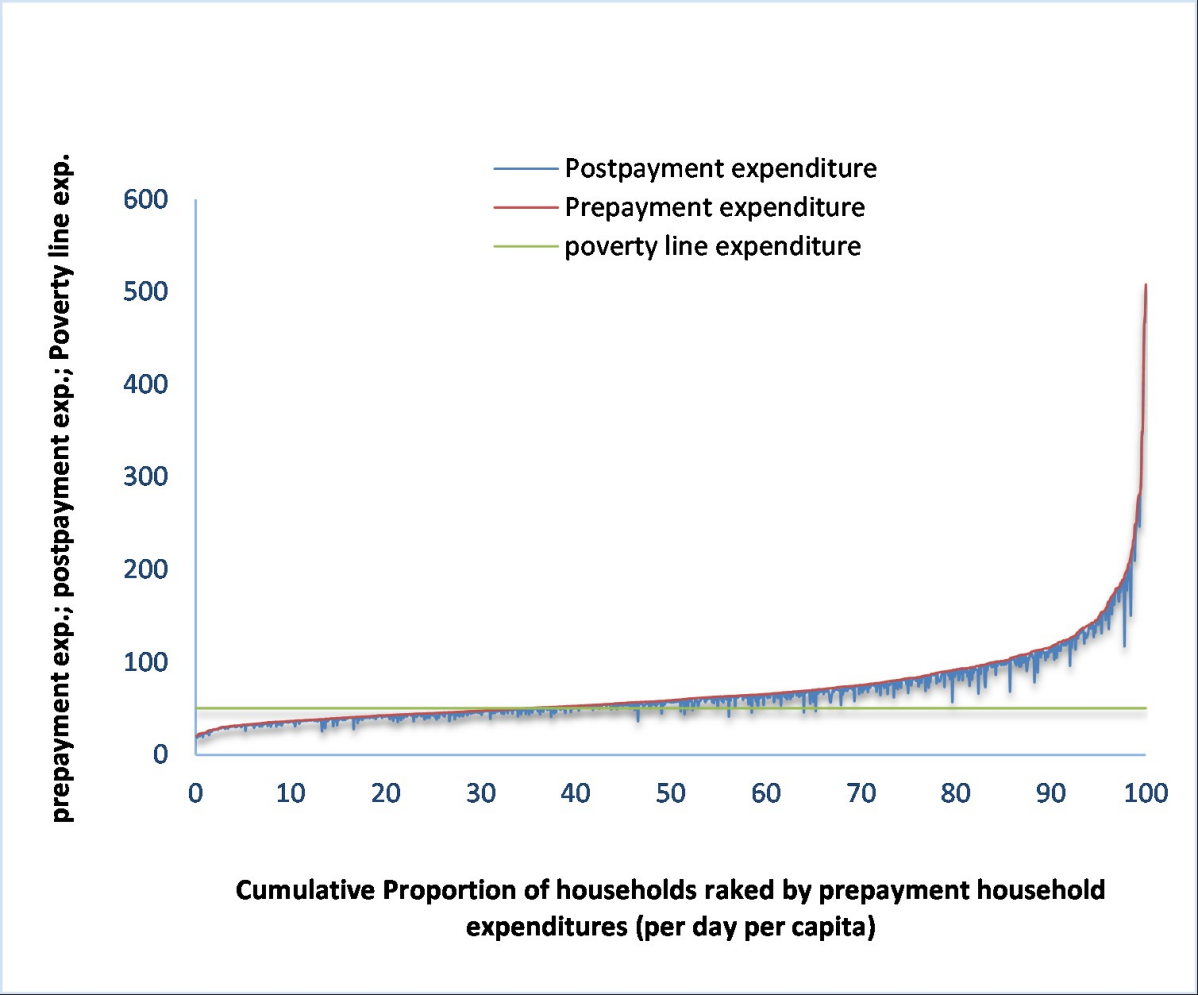
Poverty Pen’s parade graph for all ailments in HDP areas.

**Fig 3:**
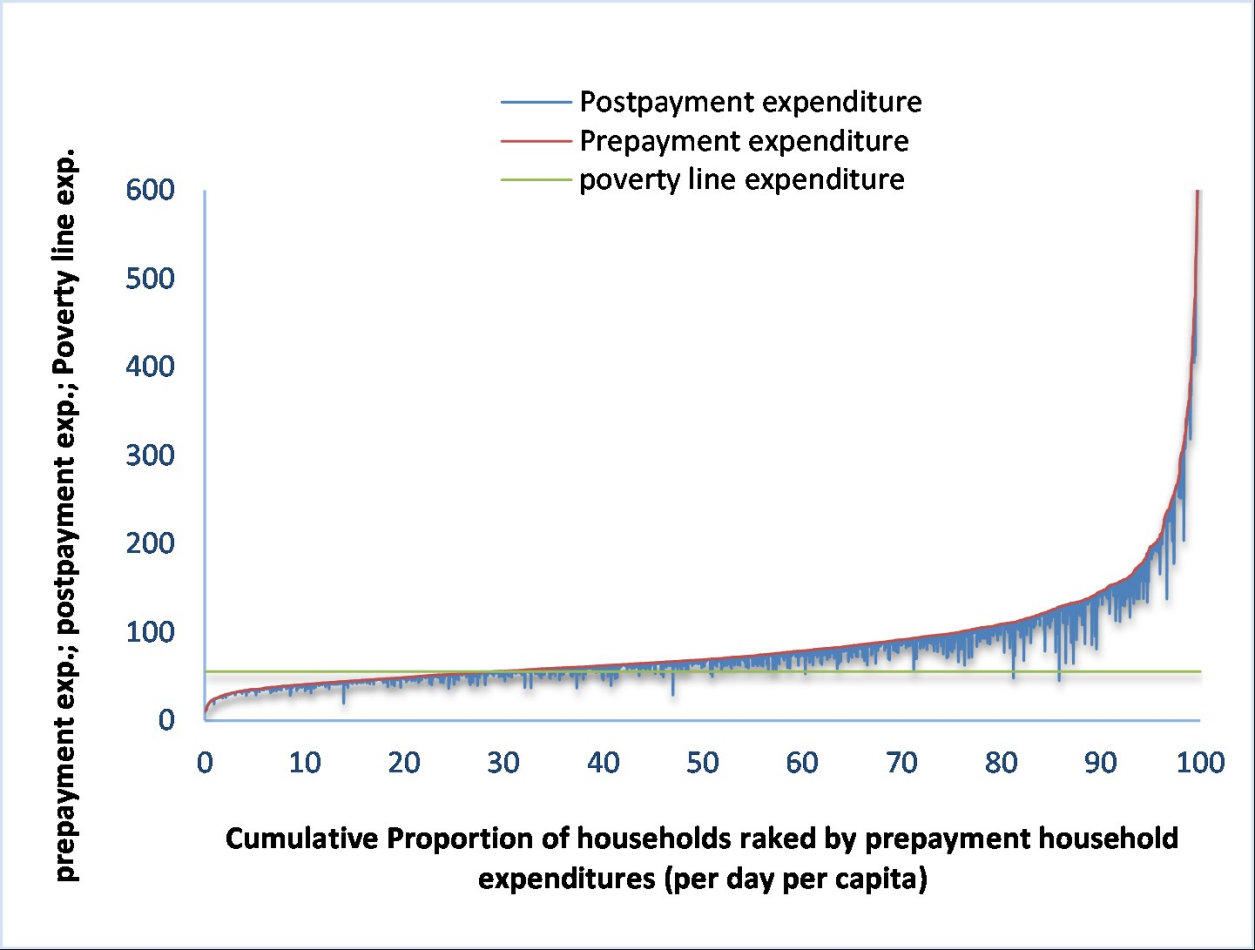
Poverty Pen’s parade graph for all ailments in LDP areas.

As seen, there was obvious variation in headcount impoverishment impact of OOP payments between HDP and LDP areas (Table 5, column 5). OOP payments dragged 16.5 percent households down into poverty in HDP areas while the corresponding figure was 11.3 percent in LDP areas. The Pen’s parade graphs also testify the same (see Figs 2 and 3). Thus, headcount impoverishment impact of OOP payments for healthcare was much higher in HDP areas compared to LDP areas. The variation in headcount impoverishment impact of OOP payments for healthcare was also explicit among the HDP areas. Out-of-pocket payments for healthcare raise the headcount poverty by 19.55 percent (6.5 percentage points) in *char*, 16.8 percent (5.94 percentage point) in *haor* and 13.1 percent (4.26 percentage points) in coastal areas.

Due to payments for health care, headcount poverty increased by about 62 percent (13.23 percentage points) among those who incurred catastrophic health expenses (Table 5, columns3-5). This impact was higher in LDP areas (69% or 12.85 percentage points) compared to HDP areas (52.7% or13.96 percentage points). Headcount impoverishment impact for catastrophic health events was highest (about 63% or 15 percentage points) in coastal areas although their incidence was highest in *char*.

**Fig 4:**
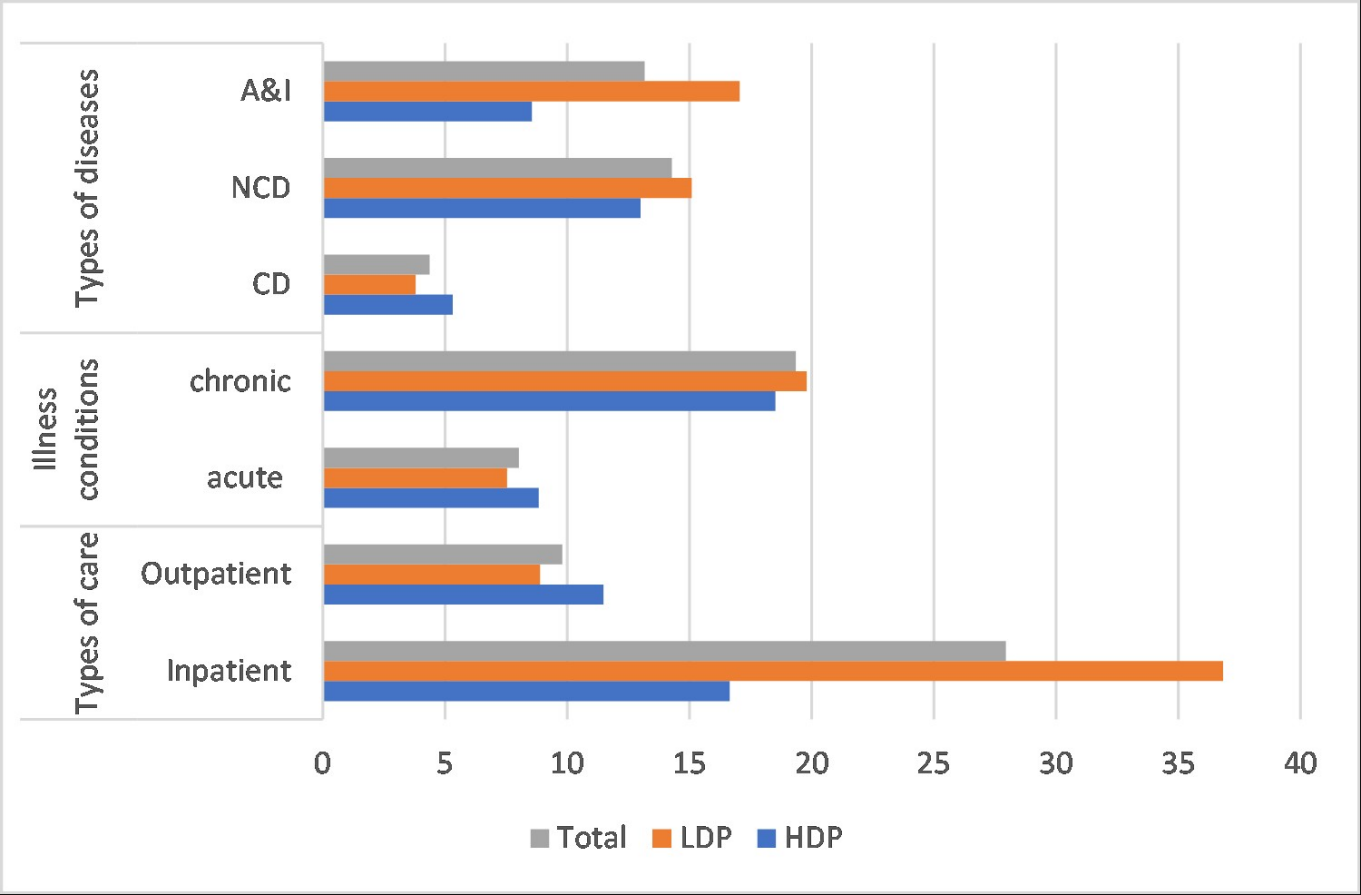
Impoverishment impact of OOP payments for healthcare by types of diseases, illness conditions and types of care in terms of relative poverty.

Tables 6 and 7 and Fig 4 illustrates the impoverishment impact of OOP payments for broad types (or conditions) of illnesses and type of cares respectively. The headcount impoverishment impact of OOP payments for CDs, NCDs and A&I was 4.4, 14.3 and 13.2 percent; for acute and chronic was about 8.0 and 19.4 percent; and for inpatient and out-patient was 28.0 and 9.8 percent respectively (Columns3-5, Tables 6 & 7).

**Table 6:** Impact of OOP payments on poverty by types of diseases/illness conditions and geographical areas.

Contents		Types of geographical areas	Poverty Headcount (in %)			Poverty Intensity (in BDT)			Poverty impact (Normalized poverty gap (in %))
			Pre-payment	Post payment	Poverty impact in percentage points^§^	Pre-payment	Post payment	Poverty impact (intensity)^§^	
						Mean [SD] (n)	Mean [SD] (n)		
Types of diseases	CDs	HDP areas	47.36 (394)	49.88 (415)	2.52 (5.32%)	11.83 [7.88] (394)	11.94 [8.07] (415)	0.11 (0.93%)	0.22
		LDP areas	38.58 (704)	40.05 (731)	1.47 (3.81%)	14.17 [10.04] (704)	14.26 [10.09] (731)	0.09 (0.64%)	0.16
		Total	41.32 (1,098)	43.13 (1,146)	1.81 (4.38%)	13.33 [9.38] (1,098)	13.42 [9.47] (1,146)	0.09 (0.68%)	0.17
	NCDs	HDP areas	40.27 (354)	45.51 (400)	5.24 (13.01%)	11.36 [7.55] (354)	11.85 [7.78] (400)	0.49 (4.31%)	0.97
		LDP areas	32.44 (570)	37.34 (656)	4.90* (15.10%)	13.33 [9.71] (570)	13.72 [10.05] (656)	0.39 (2.93%)	0.71
		Total	35.05 (774)	40.06 (1,056)	5.01** (14.29%)	12.58 [8.99] (924)	13.01 [9.29] (1,056)	0.43 (3.42%)	0.82
	I & A	HDP areas	38.04 (35)	41.30 (38)	3.26 (8.57%)	11.37 [8.71] (35)	12.10 [8.36] (38)	0.70 (6.42%)	2.39
		LDP areas	29.71 (41)	34.78 (48)	5.07 (17.06%)	11.26 [8.24] (41)	11.29 [8.57] (48)	0.03 (0.27%)	0.80
		Total	33.04 (76)	37.39 (86)	4.35 (13.17%)	11.32 [8.40] (76)	11.70 [8.45] (86)	0.34 (2.98%)	0.91
Illness conditions	Acute	HDP areas	43.03 (475)	46.83 (517)	3.80 (8.83%)	11.47 [7.83] (475)	12.10 [7.98] (517)	0.63 (5.49%)	1.25
		LDP areas	36.46 (862)	39.21 (927)	2.75 (7.54%)	13.89 [9.90] (862)	14.22 [9.96] (927)	0.33 (2.38%)	0.60
		Total	38.55 (1,337)	41.64 (1,444)	3.09* (8.02%)	13.03 [9.29] (1,337)	13.46 [9.35] (1,444)	0.43 (3.30%)	0.82
	Chronic	HDP areas	36.00 (135)	42.67 (160)	6.67 (18.53%)	10.67 [7.35] (135)	10.79 [7.69] (160)	0.12 (1.12%)	0.24
		LDP areas	28.54 (232)	34.19 (278)	5.65 (19.80%)	11.70 [9.72] (232)	12.39 [10.42] (278)	0.69 (5.90%)	1.25
		Total	30.89 (367)	36.87 (438)	5.98* (19.36%)	11.32 [8.93] (367)	11.80 [9.53] (438)	0.48 (4.24%)	0.91

Note

^§^The figure in parentheses refers the poverty impact in percentage.

***, **, and * indicates significance at 1%, 5%, and 10% respectively.

**Table 7:** Impact of OOP payments on poverty by types of cares and geographical areas.

Contents		Types of geographical areas	Poverty Headcount (in %)			Poverty Intensity (in BDT)			Poverty impact (Normalized poverty gap (in %))
			Pre-payment	Post payment	Poverty impact in percentage points^§^	Pre-payment	Post payment	Poverty impact (intensity)^§^	
						Mean [SD] (n)	Mean [SD] (n)		
Type of care	IP	HDP areas	39.74 (60)	46.36 (70)	6.62 (16.66%)	11.44 [8.14] (60)	12.55 [7.94] (70)	1.11 (9.70%)	2.21
		LDP areas	26.39 (76)	36.11 (104)	9.72 (36.83%)	11.45 [8.59] (76)	12.69 [1.22] (104)	1.24 (10.83%)	2.25
		Total	30.98 (136)	39.64 (174)	8.66 (27.95%)	11.45 [8.36] (136)	12.63 [9.35] (174)	1.18 (10.31%)	2.24
	OP	HDP areas	41.36 (479)	46.11 (534)	4.75 (11.48%)	11.50 [7.74] (479)	12.00 [7.99] (534)	0.5 (4.35%)	0.99
		LDP areas	36.06 (887)	39.27 (966)	3.21 (8.90%)	13.73 [9.93] (887)	14.09 [10.04] (966)	0.36 (2.62%)	0.65
		Total	37.76 (1,366)	41.46 (1,500)	3.7** (9.80%)	12.94 [9.28] (1366)	13.34 [9.41] (1,500)	0.4 (3.09%)	0.76

Note

^§^The figure in parentheses refers the poverty impact in percentage.

***, **, and * indicates significance at 1%, 5%, and 10% respectively.

Thus, intuitively the impoverishment impact of OOP payments for NCDs, chronic and inpatient care was much higher than their respective counterpart. The impoverishment impact of OOP payments for each type of illness and care (apart from A&I and IP) was higher (in terms of absolute poverty) in HDP areas compared to LDP areas.

For CDs the headcount impoverishment impact was highest in *haor* (about 8 percent) followed by *char* (4 percent) and coastal (3 percent). This was highest for NCDs in coastal (about 15 percent) followed by *char* (14 percent) and *haor* (12 percent). For A&I, this was highest in *haor* (14 percent) followed by *char* (10 percent) (results are not shown in Table).

The impoverishment impact for acute illnesses was highest in *haor* (about 12 percent) followed by *char* (8 percent) and coastal (7 percent) while the variation of this impact for chronic illnesses was very little among *char*, *haor* and coastal areas (16, 20 and 20 percent respectively). For inpatient care this impact was highest in *haor* (20 percent) followed by *char* (about 17 percent) and coastal (13 percent) while there was no variation between coastal *and haor* (12.7 percent for both) for outpatient care. This impact was 9.3 percent in *char* areas (results are not shown in table).

It is noticeable that, in terms of headcount impoverishment impact, households in the HDP areas were the worst affected in which *haor* areas are the most badly affected areas followed by *char*.

#### Average poverty gap

The average pre-payment and post-payment poverty gap (per day per capita) was BDT 14.95 and BDT 15.41 respectively (Table 5, columns 6–7). In other words, per day per capita expenditure before paying for health care was low by on average BDT 14.95 from the poverty line while it was BDT 15.41 after paying for health care. Thus, OOP payments for healthcare raised the poverty gap per day per capita by BDT 0.46 or by 3.08 percent (Table 5, column 8). That is, the expenditure level of the poor households’ dips by on average 3.08 percent per day per capita due to OOP payments for healthcare. It is also noticeable from the Pen’s parade graph (Fig 1), which shows the extent of poverty gap is measured by the area below the poverty line above each parade [10], that health care expenses increased the intensity of poverty. Out-of-pocket payments for healthcare raised the average poverty gap by about 3 percent in HDP areas and by 7 percent in LDP areas. This impact also varied among the HDP areas. The average poverty gap increased, due to OOP payments for healthcare, by 5.45, 2.45 and 2.30 percent in the *char*, *haor* and coastal areas respectively.

The pre-payment and post-payment (per day per capita) average poverty gap for catastrophic events was BDT 14.43 and BDT 16.28. Thus, OOP payments for healthcare increased the average poverty gap by about 13 percent for catastrophic events (Table 5, column 8). This impact was higher in HDP areas (16.95 percent) compared to LDP areas (9.51percent). There were also large variations of this impact among the HDP areas: about 19, 34 and 7 percent in *char*, *haor* and coastal respectively.

The impact of OOP payments for healthcare on the average poverty gap was evidently much higher for NCDs, chronic and inpatient care than their respective counterpart (Table 6, column 8). It is also noticeable that the impact of OOP payments for healthcare on the average poverty gap was quite higher in HDP areas than LDP areas for all but chronic illnesses and inpatient cases.

Among the HDP areas this impact is much higher in *char* areas for all kind of illnesses except inpatient care and A&I (results are not shown in Table).

#### Normalized poverty gap

Normalized poverty gap measures the size of the poverty gap in relation to the poverty line. Comparative analysis is more meaningful by using normalized poverty gap because it is independent of different types of currency or the choice of the poverty line [20]. The last column of Table 5 shows the normalized poverty gap by all ailments and catastrophic events. For all ailments, the relative burden is higher in LDP areas than HDP areas while, in case of catastrophic events, it is considerably greater in HDP areas than its counterpart. Normalized poverty gap was respectively 0.85 and 2.01 percent for all ailments in HDP and LDP areas whereas this was 4.23 and 2.74 percent for catastrophic events. Among the HDP areas, the relative burden of catastrophic healthcare expenditures was highest in *haor* areas (6.02 percent) followed by *char* (5.29 percent) and coastal (1.75 percent).

The last column of Table 6 displays the normalized poverty gap by diseases type, illness conditions and types of care. It is intuitively seen that NCDs, chronic illness and inpatient care has greater relative burden than their respective counterpart. The relative burden was higher in HDP areas than LDP areas for all but chronic illness and inpatient cases.

Among the HDP areas the relative burden is highest in *char* (13.20 percent) followed by coastal (9.89 percent) and *haor* (9.82 percent) for NCDs while corresponding figure for A&I was also highest in *char* (9.47) compared to the counterpart. For both acute and chronic conditions, the normalized poverty gap was the highest in *haor* (10.22 percent and 13.96 percent respectively) than other HDP areas. For inpatient care the normalized poverty gap was highest in *char* (15.87 percent) while this was highest in *haor* (10.88 percent) for outpatient care (results are not shown in Table).

## Discussions and conclusion

This study reveals the variation in the impoverishment impact of OOP payments between HDP areas and LDP areas using a data set of 3,791 households in rural Bangladesh. This article measures the impoverishment impact of OOP payments for health care by estimating different poverty lines specific to the regions. The broader focus of the investigation would thus be to gauge how the levels of OOP from various ailments affect the household living standard across the regions experiencing different climate change risks in Bangladesh. This paper estimated three poverty measures, such as poverty headcount, poverty intensity or gap and normalized poverty gap for all ailments, catastrophic events, diseases types (CDs, NCDs, A&I), illness conditions (acute and chronic) and hospitalization across the regions. As this study is the first of its kinds, the results are not directly comparable with the existing literature to a large extent.

The results show that about half of the sampled individuals (or almost all the sampled households) had some sort of ailments over 12 months recall period. About one in every six individuals (or more than one-third households) had at least one episodes of chronic illness over 12 months. The incidence of CDs is significantly (*p-value ≤0*.*01)* higher than the NCDs. The hospitalization care received by 5.6 percent of the patients. However, there is no significant difference in the incidence of morbidity irrespective of type of disease and illness conditions between HDP and LDP areas. However, the *char* area had remarkably high morbidity rate (about 70 percent) in the HDP areas. One of the reasons is the substantially higher incidence of injury and accidental ailments.

A household affected with any ailment over 12 months had to spend, on an average, about BDT 5,590 for health care, which was 4.6 percent of total household expenditure and 8.7 percent of the food expenses. There was no significant difference in these shares between HDP and LDP areas nevertheless the OOP payments for healthcare were significantly (*p-value≤0*.*01)* higher in LDP areas. Like morbidity rate, share of OOP payments for healthcare of household expenditure (6.9 percent) was highest in the *char* areas.

Somewhat similar evidence was found in most of the literature as far as OOP payments for healthcare as the share of total household expenditure is concerned. OOP payments for healthcare in Bangladesh, as found by a previous study [12], were 5.1percent of total household expenditure. A prior study in Bangladesh found that 5.8 percent of the total household expenditure or 9.4 percent of food expenditure was spent by the households for health care in some LDP areas of Bangladesh [51]. Another study on 11 Asian countries found that 5 percent of total household budget was spent by 28 percent households in Bangladesh (and 4.5% was spent by more than 25 percent households) on OOP health expense [52]. A study in India found that about 5 percent of total household expenditure was spent as OOP payments for healthcare [11].

Drug cost, the leading factor of OOP outlays, accounted for about 70 percent of OOP payments. Other studies also found similar evidence [51,53,54]. Drug cost was about 74 percent of OOP outlays in HDP areas (76, 75,72 percent in *char*, *haor*, and coastal areas respectively) and 68 percent in LDP areas. The greater share of OOP expense in the HDP areas accounted for drugs because of their dependency on the drugstores due to lack of healthcare facilities with proper diagnostic, inpatient care and surgical facilities.

Overall, about one in six households faced catastrophic healthcare expenditure at the 10 percent threshold level over 12 months period. The difference in catastrophic health care expenditure between HDP and LDP areas was not robust. However, the magnitude of catastrophic healthcare expenditure is significantly (*p-value≤ 0*.*05)* higher in *char* than *haor*. At the same threshold level, it was found in a previous study that 12 percent households faced catastrophic expenditure in some LDP areas in Bangladesh [14]. Similar evidence (14 percent in the national level and16 percent in the rural level) was found in another study [24]. The other study found a higher catastrophic healthcare expenditure (24.6 percent in the national level) [38]. In rural India, two different studies found about 34 percent and 15 percent households faced catastrophic healthcare expenditure [13,55]. This is to note that the existence of a community-based health insurance scheme for the latter case may be a reason for lower catastrophic health care expenditure.

In terms of relative poverty, OOP expense for health care accounted for pushing 13.1 percent (absolute poverty: 4.1 percent) households down the poverty line. The HDP areas experienced substantially higher increase in headcount impoverishment through OOP outlays compared to the LDP areas; it implies that many households of these areas were concentrated around the poverty line and consequently even a low level of OOP payments pulled down a large number of households below the poverty line. The impoverishment impact of OOP outlays for healthcare in Bangladesh, as found in earlier studies, varies from 3.8 percent to 9.6 percent in terms of relative poverty [12,14,24,37]. It was 3.24 percent (in terms of absolute poverty) in India [11] and 0.4 percent in Turkey [22]. Note that the latter used a different estimation procedure.

Catastrophic health payment is the core influencing factor for the incidence of poverty because a large number of households fell into poverty (absolute poverty: 13.23 percent and relative poverty: 61.79 percent) due to catastrophic health payments. The absolute poverty was higher in HDP areas compared to LDP areas while the relative poverty reveals the contrary fallouts. The households with NCDs, chronic illness or inpatient cases fell more into poverty through OOP payments compared to its counterpart because these factors are accountable for incurring the highest OOP outlays by the households than the other factors. The NCD related impoverishment impact (14.3 percent) was almost double than what was found in a study using national level data [26]. For all but NCDs, chronic and inpatient cases, HDP areas had a higher incidence in headcount poverty compared to the LDP areas. The higher incidence of CDs along with the paucity of treatment facilities for the aforementioned diseases in HDP areas may be the reasons behind this incongruity. It is worth mentioning that climate change risk may be a determining factor for higher incidence of CDs in HDP areas. A member in the households faced catastrophic health expenses needs to increase his/her daily expenditure on average BDT1.85 to lift out of poverty. Both absolute and relative average poverty gap were more wider in HDP than LDP areas due to catastrophic OOP outlays for health care.

The HDP than the LDP areas had substantially higher incidence of poverty despite lower OOP payments in HDP areas for the most of the cases of interest. More preciously, the population in the HDP areas, despite spending lower amount from out-of-pocket, had to experience higher impoverishment burden than LDP areas. In addition, incidence of poverty in HDP areas were exacerbated especially by the catastrophic medical expenses. Moreover, population in the HDP areas, especially *char* and *haor* are more susceptible to any level of OOP payments due to low level of earnings.

Mainly the household with catastrophic healthcare expenditure and NCDs had the higher risk of falling into poverty. These auspicious factors of the poverty incidence are also interrelated. That is, NCDs is responsible for both high OOP health outlays and catastrophic healthcare expenditure and this result is also bolster by existing literature [26,39,40]. A number of programs are already embodied in the government policy documents for NCDs prevention and control such as restricting tobacco use, increasing physical activity, encouraging to intake healthy diets, ensuring food safety and controlling air pollution [56]. Some of these measures were also recommended in some prior studies [57,58]. However, there is a lack of proper implementation and monitoring of these programs [59]. Hence, actions need to be taken for proper implementation and monitoring of these programs for reducing catastrophic expenditure and the level of impoverishment thereof through preventing the escalation of NCDs.

It is also noted that mental health has close link with NCDs and they both have many common risk factors [60]. The prior studies show that mental diseases account for high catastrophic healthcare expenditure as well as impoverishment impact [14,61]. Nevertheless, mental health is quite neglected in low- or middle-income countries [62] though it gets importance in healthcare priority setting in developed countries. Thus, improving mental health through necessary measures may also reduce the impoverishment impact.

The HDP areas, especially *char* and *haor*, are the major hard to reach areas in Bangladesh. The settlement of population is extremely scattered in the *char*. On the other hand, *haor* remains disconnected with district headquarter or even sub-district headquarter for most of the time. Absenteeism of health workforce in these areas is the major obstacle for non-functionality of public health system. Lack of private facilities along with non-functionality of public facilities compel the people to seek healthcare from informal providers, mainly drugstores, which basically sell medicines to the patients. Thus, medical care is synonymous to the consumption of medicine in HDP areas. About two-thirds share of drug costs in the OOP payments for healthcare, as found in this study, is a reflection of this claim.

Given the non-functionality of public providers and lack of private providers, there is not much option for providing better health care for the population of HDP. It is worth mentioning that the micro-health insurance is assumed to be a feasible solution, however, evidence shows that it fails to provide financial risk protection [63]. Community Clinic (CC), which has been established for every 6,000–8,000 population in the rural areas where a Community Health Care Provider (CHCP) provides health services with free medicine, may be the last resort for serving this population. However, the CCs have some major shortcoming [64]. Thus, government should give more emphasis, especially in HDP areas, on making the existing CCs more functional through providing further training to the CHCP and ensuring availability of medicine and screening equipment for early detection of NCDs. It may be also thought for establishing new CC even for lesser number of population (e.g., 2,000–3,000) especially in *char* and *haor* areas.

## Limitations

The study may have biases for using the 12 months recall period. Moreover, potential biases also be developed with the use of self-reported data.

## Supporting information

S1 File Questionnaire. (PDF)
