# Supplementary material for: Impoverishment impact of out-of-pocket payments for healthcare in rural Bangladesh: Do the regions facing different climate change risks matter?
Source: PLoS One. 2021 Jun 4;16(6):e0252706. doi: 10.1371/journal.pone.0252706 (PMC8177643; doi:10.1371/journal.pone.0252706)
Supplement: S1 File — (PDF) [file pone.0252706.s001.pdf]

Institute of Microfinance (InM)
ইনস্টিটিউট অব মাইক্রোফিন্যান্স (আইএনএম)
পিকেএসএফ ভবন, ই-৪/বি, আগারগাঁও প্রশাসনিক এলাকা, ঢাকা

Study on

Microinsurance, Poverty and Vulnerability

May-June 2011

আমি (নাম উল্লেখ করুন) একটি গবেষনা কাজের উপাত্ত সংগ্রহের জন্য ঢাকা খেকে এসেছি। আপনি জেনে খুশি হবেন যে, **ইনস্টিটিউট অব মাইক্রোফিন্যান্স** “`vwi`ª এবং ¶y`ªexgv” বিষয়ে একটি গবেষনা কর্ম পরিচালনা করছে। এই গবেষনার জন্য বাংলাদেশের ১২০টি গ্রামের ৪ হাজার পরিবারকে মনোনিত করা হয়েছে। আপনার পরিবার উক্ত ৪ হাজার পরিবারের মধ্যে একটি। এই গবেষনায় উক্ত ৪ হাজার পরিবারের পরিবারিক জীবনের বিভিন্ন সংকট, বিপদ/আপদ, রোগ-ব্যাধি ও অন্যান্য আর্থ সামাজিক অবস্থা সম্পর্কে কিছু উপাত্ত সংগ্রহ করা হবে। উল্লেখ্য যে, আপনাদের দেওয়া তথ্য শুধুমাত্র গবেষনার কাজে ব্যবহার করা হবে। আমি এ উপাত্ত সংগ্রহের কাজে আপনার সহযোগিতা কামনা করছি।

আপনি কি আপনার পরিবারের এ সকল উপাত্ত দিয়ে আমাদের গবেষনা কাজে সহযোগিতা করতে ইচ্ছুক?

1. হ্যাঁ (হ্যাঁ হলে খানা রোষ্টার সেকশনে যান)
2. না
3. পরে আসুন (সময় নিয়ে পরে যান)।

আপনাকে আনেক ধন্যবাদ।

খানা রোস্টার

খানা ID | খানা প্রধানের ভোটার ID নাম্বার | প্রশ্ন ID | বিভাজিত খানা কোড

মাঠ কর্মকর্তার নাম: | সাক্ষাৎকার গ্রহণের তারিখঃ DD . MM . YYYY

1. জেলা: | কোডঃ | 2. উপজেলা/ থানা: | কোডঃ

3. ইউনিয়ন /পৌরসভা | কোডঃ | 4. গ্রাম/ওয়ার্ড | কোডঃ

5. এরিয়া প্রোগ্রাম ☐ কন্ট্রোল ☐ | 6. খানার বিশেষ পরিচিতি ঃ

7. খানা প্রধানের নাম: | 8. ধর্ম ইসলাম ☐ হিন্দু ☐ বৌদ্ধ ☐ খ্রীষ্টান ☐ অন্যান্য ☐

9. খানা প্রধানের পিতা/স্বামীর নাম:

10. প্রধান উত্তরদাতার নাম: | সদস্য কোড

11. খানা প্রধানের সাথে সম্পর্ক ঃ (কোড-1) | 12. বাড়ীতে কি বিদ্যুৎ আছে ? হ্যাঁ ☐ না ☐ | 13. খানার উপার্জনকারী কতজন সদস্য বিদেশে আছেন?

14. কি ধরনের পানি পান করেন? আর্সেনিকমুক্ত টিউবওয়েলের পানি ☐ আর্সেনিকযুক্ত টিউবওয়েলের পানি ☐ পুকুরের/কুপের পানি ফুটিয়ে ☐ পুকুরের/কুপের পানি না ফুটিয়ে ☐ টেপের পানি ফুটিয়ে ☐ টেপের পানি না ফুটিয়ে ☐ অপরিক্ষীত টিউবয়েলের পানি ☐ অন্যান্য (উল্লেখ করুন) ☐

15. কি ধরনের পায়খানা ব্যবহার করেন? কোন পায়খানা নেই ☐ কাঁচা পায়খানা ☐ স্বাব পায়খানা ☐ পাকা পায়খানা ☐

16. সাক্ষাৎকার গ্রহণকারীর দৃষ্টিতে থাকার ঘর শুকনা ☐ স্যাঁতস্যাঁতে ☐

17. সাক্ষাৎকার গ্রহণকারীর দৃষ্টিতে থাকার ঘরের আশে পাশের পরিবেশ পরিস্কার ☐ মোটমুটি পরিস্কার ☐ নোংরা ☐ খুবই নোংরা ☐

18. বাসস্থানের/প্রধান ঘরের বর্ণনা (যেখানে সম্ভব পর্যবেক্ষণ করুন/অথবা খানা প্রধান বা তার স্ত্রী/স্বামীকে জিজ্ঞাসা করুন)। দেয়ালে ব্যবহৃত উপকরণ (কোড-2) | মেঝে তৈরীর উপকরণ (কোড-2) | ছাদের উপকরণ (কোড-2)

19. আপনার খানা হতে নিকটতম স্বাস্থ্যকেন্দ্রের বা স্বাস্থ্যসেবা প্রদানকারীর ধরন (কোড-11) | 20. আপনার খানা থেকে নিকটতম স্বাস্থ্যকেন্দ্রের দূরত্ব কত? মাইল: .

21. আপনার খানায় গত দুই বছরে কোন বিভাজন হয়েছিলো কি? হ্যাঁ ☐ না ☐ | 22. আপনার খানা গত দুই বছরে কয় ভাগে বিভাজিত হয়েছিলো?

শুধুমাত্র প্যানেল এরিয়ার জন্য 23. গ্রামীন কল্যানের স্বাস্থ্যকেন্দ্র থেকে খানার দূরত্ব কত? . | 24. গ্রামীন কল্যানের স্বাস্থ্যকেন্দ্রে যেতে কত টাকা খরচ হয়?

অন্যান্য উল্লেখ করুন

101	102	103	104	105	106	107	108	109	110	111	112	113	114	115	116
খানা সদস্য	খানা সদস্যদের নাম	লিঙ্গ<br>পুরুষ = 1<br>মহিলা = 2	বয়স (বছর) পূর্ণ বছরে লিখুন। এক বছরের নীচে হলে ‘0' বসান।	খানা প্রধানের সাথে সম্পর্ক (কোড-1)	বৈবাহিক অবস্থা<br>(কোড-3)	শিক্ষা (পাশ করা শ্রেণী) উত্তর 0 থেকে 17 লিখতে হবে* (কোড-4)	গত ১২ মাসে কেউ লেখা পড়া বাদ দিয়েছে কি?<br>হ্যাঁ =1<br>না = 2<br>(2 হলে 110 এ যান)	যদি হ্যাঁ হয় তবে লেখা পড়া বাদ দেয়ার কারন (কোড-5)	ধূমপায়ী কি না?<br>হ্যাঁ =1<br>না = 2	উপার্জন/ আয় করেন কি?<br>হ্যাঁ =1<br>না = 2	প্রধান পেশা<br>(কোড-6)	২য় পেশা<br>(কোড-6)<br>না থাকলে 0 লিখুন	খানায় অবস্থান?<br>খানায় আছেন=1<br>বিদেশে আছেন=2<br>দেশের অভ্যন্তরে অন্যত্র = 3	কি কারনে বিদেশে বা দেশের অভ্যন্তরে অন্যত্র অবস্থান করছেন? (কোড-7)	কত মাস ধরে বিদেশে বা দেশের অভ্যন্তরে অন্যত্র আছেন ?
1		1 2		খানা প্রধান=01			1 2		1 2	1 2			1 2 3		
2		1 2					1 2		1 2	1 2			1 2 3		
3		1 2					1 2		1 2	1 2			1 2 3		
4		1 2					1 2		1 2	1 2			1 2 3		
5		1 2					1 2		1 2	1 2			1 2 3		
6		1 2					1 2		1 2	1 2			1 2 3		
7		1 2					1 2		1 2	1 2			1 2 3		
8		1 2					1 2		1 2	1 2			1 2 3		
9		1 2					1 2		1 2	1 2			1 2 3		
10		1 2					1 2		1 2	1 2			1 2 3		
11		1 2					1 2		1 2	1 2			1 2 3		
12		1 2					1 2		1 2	1 2			1 2 3		
13		1 2					1 2		1 2	1 2			1 2 3		
14		1 2					1 2		1 2	1 2			1 2 3		
15		1 2					1 2		1 2	1 2			1 2 3		
16		1 2					1 2		1 2	1 2			1 2 3		
17		1 2					1 2		1 2	1 2			1 2 3		

117. আপনার পরিবারের প্রধান সিদ্ধান্ত গ্রহীতা কে? সদস্য কোড

অন্যান্য উল্লেখ করুন

সেকশন-2: সংকট ও মোকাবেলা (গত দুই বছরে নিম্নোক্ত কোন সংকটের শিকার হয়ে থাকলে তার বিবরণ।) *প্যানেলের জন্য ২০০৯ সালের আগস্ট মাস থেকে আজ পর্যন্ত

201. মৃত্যু সংক্রান্ত সংকট

1. আপনার খানায় গত দুই বছরে কোন সদস্য মৃত্যুবরন করেছেন কি? হ্যাঁ ☐ না ☐

	2	3	4	5	6	7	8		9		10		11				12	13	14	15
SN	সংকটের ধরণ (মৃত্যু)	মৃত্যুর সময় মৃত ব্যক্তির (পূর্ন বছর)	মৃত ব্যক্তির লিঙ্গ পুরুষ = 1 মহিলা = 2	মৃত্যুর আগে মৃত ব্যক্তি উপার্জন করতেন কি? হ্যাঁ =1 না = 2 (না হলে 8 নং প্রশ্নে যান)	মৃত্যুর আগে মৃত ব্যক্তির পেশা (কোড-6)	মৃত্যুর আগে মৃত ব্যক্তির গড় মাসিক আয় (টাকা)	মৃত্যুবরনের কারণ (কোড-8)		মৃত্যুর পূর্বে চিকিৎসা বাবদ খরচ আর্থিক ক্ষতি হিসেবে বিবেচনা করে মোট আর্থিক ক্ষতি (টাকা) উল্লেখ করুন		মৃত ব্যক্তির দাফন-কাফন/সৎকার এবং সামাজিক অনুষ্ঠানাদি বাবদ খরচ (টাকা) (যদি প্রযোজ্য হয়) (দূর্ঘটনায় মৃত্যু হলে লাশ খোজাখুজির খরচ)		9 এবং 10 তে উল্যেখিত খরচ কিভাবে মোকাবেলা করেছিলেন? (প্রধান দুটি উৎস উল্লেখ করুন) (কোড-9)				মৃত ব্যক্তির কোন জীবন বীমা ছিলো কি? হ্যাঁ =1 না = 2	বীমার দাবী উত্থাপন করেছিলেন কি? হ্যাঁ =1 না = 2	বীমা থেকে পাওয়া টাকার পরিমান (টাকা) (প্রক্রিয়াধীন হলে 1 লিখুন)	বীমা থেকে পাওয়া টাকার পরিমান পর্যাপ্ত ছিল কি? (কোড-10)
							1*	2	1*	2	1*	2	1*		2					
													উৎস 1	উৎস 2	উৎস 1	উৎস 2				
1	পরিবারের উপার্জনকারী সদস্যের মৃত্যু-1		1 2	1 2													1 2	1 2		
2	পরিবারের উপার্জনকারী সদস্যের মৃত্যু-2		1 2	1 2													1 2	1 2		
3	পরিবারের অন্য কোন সদস্যের মৃত্যু-1		1 2	1 2													1 2	1 2		
4	পরিবারের অন্য কোন সদস্যের মৃত্যু-2		1 2	1 2													1 2	1 2		

*গত ১২ মাস =1, গত ১২ মাসের আগের ১২ মাস =2। ** শুধু প্যানেলের জন্য (২০১০ এর সেপ্টেম্বর থেকে এখন পর্যন্ত=1 এবং ২০০৯ এর সেপ্টেম্বর থেকে ২০১০ এর আগস্ট পর্যন্ত=2)

202. স্বাস্থ্য সংক্রান্ত সংকট

1. আপনার খানায় বসবাসকারী সদস্যদের মধ্যে গত দুই বছরে কোন স্বাস্থ্য সংক্রান্ত সংকট হয়েছিলো কি ? (মৃত্যু ব্যতীত) হ্যাঁ ☐ না ☐

	2	3	4	5	6		7		8		9		10				11	12	13	14
SN	সংকটের ধরণ (স্বাস্থ্য)	সদস্য কোড	কতবার সংকট হয়েছিল?	কি রোগে ভুগেছিলেন? (কোড-13)	মোট কত দিন রোগে ভুগেছেন? (দীর্ঘস্থায়ী রোগের ক্ষেত্রে কত দিন তীব্রভাবে ভুগেছেন?)		অসুস্থ হবার আগে গড় মাসিক আয় (টাকা) (যদি প্রযোজ্য হয়)		উপার্জন থেকে কতদিন বিরত/ছুটিতে ছিলেন?		রোগ মোকাবেলার চিকিৎসা বাবদ মোট খরচ (টাকা)		কিভাবে চিকিৎসা খরচ জোগাড় করেছিলেন? (প্রধান দুটি উৎস উল্লেখ করুন) (কোড-9)				কোন স্বাস্থ্য বীমা ব্যবস্থা গ্রহন করেছিলেন কি? হ্যাঁ =1 না = 2	বীমার দাবী উত্থাপন করেছিলেন কি? হ্যাঁ =1 না = 2	বীমা থেকে পাওয়া টাকার পরিমান (টাকা) (প্রক্রিয়াধীন হলে 1 লিখুন)	বীমা থেকে পাওয়া টাকার পরিমান পর্যাপ্ত ছিল কি? (কোড-10)
					1*	2	1*	2	1*	2	1*	2	1*		2					
													উৎস 1	উৎস 2	উৎস 1	উৎস 2				
1	পরিবারের উপার্জনকারী সদস্যের দীর্ঘমেয়াদী রোগ ভোগ -1																1 2	1 2		
2	পরিবারের উপার্জনকারী সদস্যের দীর্ঘমেয়াদী রোগ ভোগ -2																1 2	1 2		
3	পরিবারের অন্যান্য সদস্যের দীর্ঘমেয়াদী রোগ ভোগ -1																1 2	1 2		
4	পরিবারের অন্যান্য সদস্যের দীর্ঘমেয়াদী রোগ ভোগ -2																1 2	1 2		
5	পরিবারের অন্যান্য সদস্যের দীর্ঘমেয়াদী রোগ ভোগ -3																1 2	1 2		
6	পরিবারের উপার্জনকারী সদস্যের স্বল্প মেয়াদী বড় ধরনের অসুস্থতা জনিত খরচ -1																1 2	1 2		
7	পরিবারের উপার্জনকারী সদস্যের স্বল্প মেয়াদী বড় ধরনের অসুস্থতা জনিত খরচ -2																1 2	1 2		
8	পরিবারের অন্যান্য সদস্যের স্বল্প মেয়াদী বড় ধরনের অসুস্থতা জনিত খরচ -1																1 2	1 2		
9	পরিবারের অন্যান্য সদস্যের স্বল্প মেয়াদী বড় ধরনের অসুস্থতা জনিত খরচ-2																1 2	1 2		

*গত ১২ মাস =1, গত ১২ মাসের আগের ১২ মাস =2। ** শুধু প্যানেলের জন্য (২০১০ এর সেপ্টেম্বর থেকে এখন পর্যন্ড়=1 এবং ২০০৯ এর সেপ্টেম্বর থেকে ২০১০ এর আগস্ট পর্যন্ত=2)

অন্যান্য উল্লেখ করুন

203. দূর্ঘটনা সংক্রান্ত সংকট 1. আপনার খানায় বসবাসকারী সদস্যদের মধ্যে গত দুই বছরে কোন দূর্ঘটনা সংক্রান্ত সংকট হয়েছিলো কি? (মৃত্যু ব্যতীত) হ্যাঁ ☐ না ☐

	2	3	4	5		6		7		8		9		10				11	12	13	14
SN	সংকটের ধরণ (দূর্ঘটনা)	সদস্য কোড	কতবার সংকট হয়েছিল?	দূর্ঘটনা ঘটার পূর্বে গড় মাসিক আয় (টাকা)		কতদিন ভুগেছিলেন?		উপার্জন থেকে কতদিন বিরত/ ছুটিতে ছিলেন?		দূর্ঘটনার ধরন উল্লেখ করুন (কোড-8)		চিকিৎসা বাবদ মোট খরচ (টাকা) (যদি প্রযোজ্য হয়)		**কিভাবে সংকট মোকাবেলা করেছিলেন? (প্রধান দুটি উৎস উল্লেখ করুন) (কোড-9)**				ঝুঁকি মোকাবিলার জন্য কোন বীমা গ্রহন করেছিলেন কি? হ্যাঁ =1 না = 2	বীমার দাবী উথাপন করেছিলেন কি? হ্যাঁ =1 না = 2	বীমা থেকে পাওয়া টাকার পরিমান (টাকা) (প্রক্রিয়াধীন হলে 1 লিখুন)	বীমা থেকে পাওয়া টাকার পরিমান পর্যাপ্ত ছিল কি? (কোড-10)
				1*	2	1*	2	1*	2	1*	2	1*	2	1*		2					
														উৎস 1	উৎস 2	উৎস 1	উৎস 2				
1	পরিবারের উপার্জনকারী সদস্য দূর্ঘটনায় আক্রান্ত-1																	1 2	1 2		
2	পরিবারের উপার্জনকারী সদস্য দূর্ঘটনায় আক্রান্ত-2																	1 2	1 2		
3	পরিবারের উপার্জনকারী সদস্য দূর্ঘটনায় আক্রান্ত-3																	1 2	1 2		
4	পরিবারের অন্যান্য সদস্য দূর্ঘটনায় আক্রান্ত-1																	1 2	1 2		
5	পরিবারের অন্যান্য সদস্য দূর্ঘটনায় আক্রান্ত-2																	1 2	1 2		
6	পরিবারের অন্যান্য সদস্য দূর্ঘটনায় আক্রান্ত-3																	1 2	1 2		

*গত ১২ মাস =1, গত ১২ মাসের আগের ১২ মাস =2। ** শুধু প্যানেলের জন্য (২০১০ এর সেপ্টেম্বর থেকে এখন পর্যন্ত=1 এবং ২০০৯ এর সেপ্টেম্বর থেকে ২০১০ এর আগস্ট পর্যন্ত=2)

অন্যান্য উল্লেখ করুন

সেকশন-2 (ক্রমশ) 204. অন্যান্য সংকট ও মোকাবেলা (গত দুই বছরে) 1. আপনার খানায় গত দুই বছরে নিম্নোক্ত কোন সংকট হয়েছিলো কি? হ্যাঁ ☐ না ☐

	1	2	3		4		5				6	7	8	9
সংকট কোড	সংকটের ধরণ (অন্যান্য)	কতবার সংকট হয়েছিল?	মোট আর্থিক ক্ষতি (টাকা) (যদি প্রযোজ্য হয়) ক্ষতিগ্রস্থ সম্পদের বাজারমূল্য উল্লেখ করুন ।		মোকাবেলার মোট খরচ (টাকা) (যদি প্রযোজ্য হয়)		কিভাবে মোকাবেলার খরচ জোগাড় করেছিলেন? (প্রধান দুটি উৎস উল্লেখ করুন) (কোড-9)				ঝুঁকি মোকাবিলার জন্য কোন বীমা জাতীয় ব্যবস্থা গ্রহন করেছিলেন কি? হ্যাঁ =1 না =2	বীমার দাবী উত্থাপন করেছিলেন কি? হ্যাঁ =1 না =2	বীমা থেকে পাওয়া টাকার পরিমান (টাকা) (প্রক্রিয়াধীন হলে 1 লিখুন)	বীমা থেকে পাওয়া টাকার পরিমান পর্যাপ্ত ছিল কি? (কোড-10)
			1*	2	1*	2	1*		2					
							উৎস 1	উৎস 2	উৎস 1	উৎস 2				
1	ফসলের ক্ষয়ক্ষতি — প্রথম পর্যায়										1 2	1 2		
	ফসলের ক্ষয়ক্ষতি — মাঝ থেকে শেষ পর্যায়										1 2	1 2		
2	ডাকাতি/চুরি										1 2	1 2		
3	গবাদি পশু মারা যাওয়া										1 2	1 2		
4	পোলট্রি ফার্মের/ মাছের খামারে ক্ষয়ক্ষতি										1 2	1 2		
5	নদী ভাঙ্গনে ঘর/জমির ক্ষয়ক্ষতি										1 2	1 2		
6	বন্যা/ঘূর্ণিঝড়ের কারনে ক্ষতি (ঘর/ভিটাবাড়ী)										1 2	1 2		
7	আগুনে ঘর পুড়ে যাওয়া										1 2	1 2		
8	দূর্ঘটনার কারণে যন্ত্রপাতি নষ্ট হওয়া										1 2	1 2		
9	ব্যবসায় লোকসান										1 2	1 2		
10	যৌতুক সংক্রান্ত খরচ***										1 2	1 2		
11	চাকুরী থেকে ছাটাই										1 2	1 2		
12	বিদেশ গমনে প্রতারণার স্বীকার										1 2	1 2		
13	মামলা-মোকদ্দমা										1 2	1 2		
14	সহিংসতা										1 2	1 2		
15	অন্যান্য (উল্লেখ করুন)										1 2	1 2		

*গত ১২ মাস =1, গত ১২ মাসের আগের ১২ মাস =2। ** শুধু প্যানেলের জন্য (২০১০ এর সেপ্টেম্বর থেকে এখন পর্যন্ত=1 এবং ২০০৯ এর সেপ্টেম্বর থেকে ২০১০ এর আগস্ট পর্যন্ত =2)। * **যৌতুকের ক্ষেত্রে আর্থিক ক্ষতি মোকাবেলার উৎসে উলেখ করুন।

অন্যান্য উল্লেখ করুন

সেকশন 3. খানার সদস্যদের গত এক বছরে রোগ ব্যাধি ও চিকিৎসা সংক্রান্ত বিষয়াদি (খানা প্রধানকে জিজ্ঞাসা করুন)

301. গত ১২ মাসে আপনার খানার কোন সদস্য কোন রোগে ভুগেছেন কি? ☐ হ্যাঁ (মৃত্যু ব্যতীত) (প্রতিটি অসুস্থতার জন্য পৃথক সারি পূরণ করুন) ☐ না (4 নং সেকশনে যান)

302	303		304	305	306	307	308	309	310	311	312	313	314	315	316	317	318
সদস্য কোড	অসুস্থতার সময়ে কি কি লক্ষন পরিলক্ষিত হয়েছিলো? (প্রধান দুটি লক্ষন) (কোড-12)		কি রোগে ভুগেছেন? (কোড-13)	কতবার অসুস্থ হয়েছিলেন?	এখন থেকে কতদিন আগে অসুস্থ হয়েছিলেন?	মোট কত দিন রোগে ভুগেছেন? (দীর্ঘস্থায়ী রোগের ক্ষেত্রে গত 12 মাসে কত দিন তীব্রভাবে ভুগেছেন?)	অসুস্থতার কারনে, উপার্জন থেকে কত দিন বিরত/ছুটিতে ছিলেন? (বিরত না থাকলে 0 লিখুন)	অসুস্থতার তীব্রতা (কোড-14)	স্বাস্থ্য সেবা গ্রহণ করেছিলেন কিনা? 1=হ্যাঁ 2= না (হ্যাঁ হলে 312 নং প্রশ্নে যান)	310 এর উত্তর না হলে, কেন স্বাস্থ্যসেবা গ্রহন করেননি? (প্রধান কারণ) (কোড-15) (317 নং প্রশ্নে যান)	310 এর উত্তর হ্যাঁ হলে এই রোগের জন্য মোট কতজন সেবাদানকারীর কাছ থেকে সেবা নিয়েছিলেন?	প্রথম কার কাছ থেকে সেবা নিয়েছিলেন? (কোড-11)	দ্বিতীবার কার কাছ থেকে সেবা নিয়েছিলেন? (কোড-11)	তৃতীয়বার কার কাছ থেকে সেবা নিয়েছিলেন? (কোড-11)	চতুর্থবার কার কাছ থেকে সেবা নিয়েছিলেন? (কোড-11)	রোগ থেকে পুরোপুরি সুস্থ্য হয়েছিলেন কি? 1=হ্যাঁ 2= না	আপনার সমবয়স্কদের লোকের তুলনায় বর্তমানে আপনার স্বাস্থ্য কেমন বলে মনে করেন? (কোড-16)
	লক্ষন 1	লক্ষন 2															
									1 2							1 2	
									1 2							1 2	
									1 2							1 2	
									1 2							1 2	
									1 2							1 2	
									1 2							1 2	
									1 2							1 2	
									1 2							1 2	
									1 2							1 2	
									1 2							1 2	
									1 2							1 2	
									1 2							1 2	
									1 2							1 2	
									1 2							1 2	
									1 2							1 2	

অন্যান্য উল্লেখ করুন

3. (ক্রমশ) প্রথম সেবাদানকারীর সম্পর্কে তথ্য

302 সদস্য কোড	304 কি রোগে ভুগেছেন? (কোড-13)	319 প্রথম সেবাদানকারীর কাছ থেকে কতবার সেবা নিয়েছিলেন?	320 প্রথম সেবাদানকারীর কাছ থেকে সেবা নেওয়ার প্রধান কারণ কি? (কোড-17)	321 সেবাদানকারীর/ সেবাকেন্দ্রের কাছ থেকে খানার দূরত্ব কতটুকু? (মাইল)	322 প্রথম সেবাদানকারীর কাছ থেকে সেবা নেওয়ার জন্য হাসপাতালে ভর্তি হতে হয়েছিলো কি? 1=হ্যাঁ 2= না	323 এই রোগের জন্য আপনাকে মোট কতদিন হাসপাতালে থাকতে হয়েছিলো?	324 পরামর্শ খরচ	325 ঔষধ খরচ	326 পরীক্ষা খরচ	327 অপারেশন খরচ	328 হাসপাতাল/ ক্লিনিক খরচ	329 যাতায়াত খরচ	330 প্যাকেজ খরচ	331 অন্যান্য খরচ	332 চিকিৎসা খরচ কিভাবে জোগাড় করেছেন? (প্রধান দুইটি উৎস) (কোড-9)				333 প্রথম সেবাদানকারীর কাছ হতে প্রাপ্ত সুবিধা দ্বারা আপনি কি সন্তুষ্ট? (কোড-18)
															উৎস 1		উৎস 2		
															উৎস	পরিমান	উৎস	পরিমান	
				.	1 2														
				.	1 2														
				.	1 2														
				.	1 2														
				.	1 2														
				.	1 2														
				.	1 2														

3. (ক্রমশ) দ্বিতীয় সেবাদানকারীর সম্পর্কে তথ্য

302 সদস্য কোড	304 কি রোগে ভুগেছেন? (কোড-13)	334 দ্বিতীয় সেবাদানকারীর কাছ থেকে কতবার সেবা নিয়েছিলেন?	335 দ্বিতীয় সেবাদানকারীর কাছ থেকে সেবা নেওয়ার প্রধান কারণ কি? (কোড-17)	336 সেবাদানকারীর/ সেবাকেন্দ্রের কাছ থেকে খানার দূরত্ব কতটুকু? (মাইল)	337 দ্বিতীয় সেবাদানকারীর কাছ থেকে সেবা নেওয়ার জন্য হাসপাতালে ভর্তি হতে হয়েছিলো কি? 1=হ্যাঁ 2= না	338 এই রোগের জন্য আপনাকে মোট কতদিন হাসপাতালে থাকতে হয়েছিলো?	339 পরামর্শ খরচ	340 ঔষধ খরচ	341 পরীক্ষা খরচ	342 অপারেশন খরচ	343 হাসপাতাল /ক্লিনিক খরচ	344 যাতায়াত খরচ	345 প্যাকেজ খরচ	346 অন্যান্য খরচ	347 চিকিৎসা খরচ কিভাবে জোগাড় করেছেন? (প্রধান দুইটি উৎস) (কোড-9)				348 দ্বিতীয় সেবাদানকারীর কাছ হতে প্রাপ্ত সুবিধা দ্বারা আপনি কি সন্তুষ্ট? (কোড-18)
															উৎস 1		উৎস 2		
															উৎস	পরিমান	উৎস	পরিমান	
				.	1 2														
				.	1 2														
				.	1 2														
				.	1 2														
				.	1 2														
				.	1 2														
				.	1 2														

অন্যান্য উল্লেখ করুন

সেকশন 3. (ক্রমশ) তৃতীয় সেবাদানকারীর সম্পর্কে তথ্য

302	304	349	350	351	352	353	354	355	356	357	358	359	360	361	362				363
সদস্য কোড	কি রোগে ভুগেছেন? (কোড-13)	তৃতীয় সেবাদানকারীর কাছ থেকে কুবার সেবা নিয়েছিলেন?	তৃতীয় সেবাদানকারীর কাছ থেকে সেবা নেওয়ার প্রধান কারণ কি? (কোড-17)	সেবাদানকারীর/ সেবাকেন্দ্রের কাছ থেকে খানার দূরত্ব কতটুকু? (মাইল)	তৃতীয় সেবাদানকারীর কাছ থেকে সেবা নেওয়ার জন্য হাসপাতালে ভর্তি হতে হয়েছিলো কি? 1=হ্যাঁ 2=না	এই রোগের জন্য আপনাকে মোট কতদিন হাসপাতালে থাকতে হয়েছিলো?	পরামর্শ খরচ	ঔষধ খরচ	পরীক্ষা খরচ	অপারেশন খরচ	হাসপাতাল /ক্লিনিক খরচ	যাতায়াত খরচ	প্যাকেজ খরচ	অন্যান্য খরচ	চিকিৎসা খরচ কিভাবে জোগাড় করেছেন? (প্রধান দুইটি উৎস) (কোড-9)				তৃতীয় সেবাদানকারীর কাছ হতে প্রাপ্ত সুবিধা দ্বারা আপনি কি সন্তুষ্ট? (কোড-18)
															উৎস 1		উৎস 2		
															উৎস	পরিমান	উৎস	পরিমান	
				.	1 2														
				.	1 2														
				.	1 2														
				.	1 2														
				.	1 2														
				.	1 2														
				.	1 2														

সেকশন 3. (ক্রমশ) চতুর্থ সেবাদানকারীর সম্পর্কে তথ্য

302	304	364	365	366	367	368	369	370	371	372	373	374	375	376	377				378
সদস্য কোড	কি রোগে ভুগেছেন? (কোড-13)	চতুর্থ সেবাদানকারীর কাছ থেকে কতবার সেবা নিয়েছিলেন?	চতুর্থ সেবাদানকারীর কাছ থেকে সেবা নেওয়ার প্রধান কারণ কি? (কোড-17)	সেবাদানকারীর/ সেবাকেন্দ্রের কাছ থেকে খানার দূরত্ব কতটুকু? (মাইল)	চতুর্থ সেবাদানকারীর কাছ থেকে সেবা নেওয়ার জন্য হাসপাতালে ভর্তি হতে হয়েছিলো কি? 1=হ্যাঁ 2=না	এই রোগের জন্য আপনাকে মোট কতদিন হাসপাতালে থাকতে হয়েছিলো?	পরামর্শ খরচ	ঔষধ খরচ	পরীক্ষা খরচ	অপারেশন খরচ	হাসপাতাল/ ক্লিনিক খরচ	যাতায়াত খরচ	প্যাকেজ খরচ	অন্যান্য খরচ	চিকিৎসা খরচ কিভাবে জোগাড় করেছেন? (প্রধান দুইটি উৎস) (কোড-9)				চতুর্থ সেবাদানকারীর কাছ হতে প্রাপ্ত সুবিধা দ্বারা আপনি কি সন্তুষ্ট? (কোড-18)
															উৎস 1		উৎস 2		
															উৎস	পরিমান	উৎস	পরিমান	
				.	1 2														
				.	1 2														
				.	1 2														
				.	1 2														
				.	1 2														
				.	1 2														
				.	1 2														

অন্যান্য উল্লেখ করুন

সেকশন-4: (সন্তান প্রসব সংক্রান্ত তথ্য)

401.আপনার খানার কেউ কি দেশে অবস্থানকালে গত ১২ মাসের মধ্যে সন্তান প্রসব করেছিলেন? (জীবিত বা মৃত) হ্যাঁ ☐ না ☐ (না হলে 5 নং সেকশনে যান) (জন্মদানকারী মাকে বা মায়ের উপস্থিতিতে এই প্রশ্নগুলো করতে হবে)

402	403	404	405	406	407		409	410	411	412	413	414		415	416		417	418		419	420
সদস্য কোড	স্বামীর সদস্য কোড **	কততম সন্তান?	এই গর্ভকালীন সময়ে কি কোন চেক আপ করিয়েছিলেন? 1=হ্যাঁ, 2=না (না হলে 409 নং প্রশ্নে যান)	হ্যাঁ হলে, কাকে দেখিয়েছিলেন? (কোড-19)	সেবাকেন্দ্র /সেবাদানকারী থেকে আপনার খানার দূরত্ব কতটুকু? (মাইল)	এই গর্ভকালীন সময়ে কতবার মেডিকেল চেকআপ করিয়েছেন?	কেন মেডিকেল চেকআপ করেননি? (প্রধান কারন) (কোড-20)	এই গর্ভকালীন সময়ে কুবার TT ইনজেকশন নিয়েছিলেন?	গর্ভকালীন সময়ে আল্ট্রাসনোগ্রাফী করানো হয়েছিলো কি? 1. হ্যাঁ 2. না (না হলে 413 নং প্রশ্নে যান)	হ্যাঁ হলে কোথায় করেছিলেন? (কোড-21)	এই গর্ভধারনের পূর্বে কখনো গর্ভধারনজনিত কোন জটিলতার সম্মুখীন হয়েছিলেন কি? 1. হ্যাঁ 2. না (না হলে 415 নং প্রশ্নে যান)	হ্যাঁ হলে, কি ধরনের গর্ভধারনজনিত জটিলতার সম্মুখীন হয়েছিলেন ? (কোড-22)		এই গর্ভকালীন সময়ে সন্তান প্রসবের পূর্বে আপনি কি কোন ধরনের জটিলতার সম্মুখিন হয়েছিলেন? 1. হ্যাঁ 2. না (না হলে 415 নং প্রশ্নে যান)	হ্যাঁ হলে, কি ধরনের গর্ভধারনজনিত জটিলতার সম্মুখীন হয়েছিলেন ? (কোড-22)		কোথায় সন্তান প্রসব করেছিলেন? (কোড-23)	কাথায় সন্তান প্রসব করতে হবে এ বিষয়ে কে সিদ্ধান্ত নিয়েছিলেন? (কোড-24)		সন্তান প্রসবের সময় কে উপস্থিত ছিল? (কোড-19)	কিভাবে সন্তান প্রসব হয়েছিলো? (কোড-25)
												1	2		1	2		1	2		
			1 2		.				1 2		1 2			1 2							
			1 2		.				1 2		1 2			1 2							
			1 2		.				1 2		1 2			1 2							

**স্বামী খানার সদস্য না হলে স্বামীর শিক্ষাগত যোগ্যতা, পেশা এবং মাসিক আয় নোট করে আনতে হবে।

* গর্ভকালীন সময় থেকে ডেলিভারী পর্যন্ত যত খরচ হয়েছে তা উল্লেখ করুন

421	422	423	424	425	426	427	428	429		430	431	432	433	434	435	436
পরামর্শ খরচ	ঔষধ খরচ	পরীক্ষা খরচ	অপারেশন খরচ	হাসপাতাল /ক্লিনিক খরচ	যাতায়াত খরচ	প্যাকেজ খরচ	অন্যান্য খরচ	চিকিৎসা খরচ কিভাবে জোগাড় করেছেন? (কোড-9) (প্রধান দুইটি উৎস)		সন্তান প্রসবের সময়ে আপনি কি কোন ধরনের জটিলতার সম্মুখিন হয়েছিলেন ? 1. হ্যাঁ 2. না (না হলে 432 নং প্রশ্নে যান)	হ্যাঁ হলে, কি ধরনের জটিলতার সম্মুখীন হয়েছিলেন ? (কোড-22)	আপনি কি মনে করেন গর্ভকালীন সময়ে স্বাস্থ্য পরীক্ষা করা উচিত? 1. হ্যাঁ 2. না	আপনি কি মনে করেন গর্ভকালীন সময়ে অতিরিক্ত/বিশেষ খাবার খাওয়া উচিত? 1. হ্যাঁ 2. না	আপনি কি কখনো আপনার স্বামীর সাথে পরিবার পরিকল্পনা বিষয়ে আলোচনা করেছেন? (কোড-26)	আপনার উপার্জিত অর্থ খরচের ওপর আপনার কি কোন নিয়ন্ত্রন রয়েছে? 1. হ্যাঁ 2. না (উপার্জন না করে থাকলে পরবর্তী সেকশনে যান)	যদি হ্যা হয়, তবে কত ভাগ? (%)
								উৎস 1	উৎস 2							
										1 2		1 2	1 2		1 2	.
										1 2		1 2	1 2		1 2	.
										1 2		1 2	1 2		1 2	.

অন্যান্য উল্লেখ করুন

আপনারা সবাই জানেন যে, বীমা মানে কিছু টাকা কিস্তিতে জমা দেয়ার মাধ্যমে ভবিষ্যতের অর্থনৈতিক বিপর্যয়ের ঝুঁকি কমানো। **501.** আপনি কি বীমা বা **Insurance** সম্পর্কে শুনেছেন? হ্যাঁ 1 না 2 (*উত্তরদাতাকে বীমা সম্পর্কে নিম্নের ধারনাগুলো দিন)

502. বীমা সম্পর্কে কিছু তথ্য দিন

1	2	3	4	5	6	7	8	9	10	11	12	13
ক্রমিক নং	বীমার ধরণ	কোন কোনটি সম্পর্কে শুনেছেন (শুনে থাকলে 1 লিখুন না হলে 0 লিখুন)	আপনার খানার কারো নিম্নোলিখিত কোন বীমা আছে/ছিলো কি? (থাকলে 1 লিখুন, না হলে 0 লিখুন)	বীমাকারীর সদস্য কোড	বীমার পরিমাণ (টাকা)	প্রতি কিস্তি বাবদ টাকার পরিমান (টাকা)	মোট কিস্তির পরিমান (সংখ্যা)	বীমা/পলিসির মেয়াদকাল (বছর)	বীমাপ্রদানকারী প্রতিষ্ঠান (কোড 27)	কখনও বীমার দাবী উত্থাপন করেছিলেন কি? হ্যাঁ=1 না=2	হ্যাঁ হলে ঐ বাবদ কোন অর্থ পেয়েছিলেন কি? হ্যাঁ=1 না=2 প্রক্রিয়াধীন=3	হ্যাঁ হলে কত টাকা পেয়েছিলেন ?
1	জীবন বীমা*	1 0	1 0							1 2	1 2 3	
2	দূর্ঘটনা (পঙ্গুত্ব) বীমা*	1 0	1 0							1 2	1 2 3	
3	স্বাস্থ্য বীমা	1 0	1 0							1 2	1 2 3	
4	সম্পদ বীমা*	1 0	1 0							1 2	1 2 3	
5	ফসল বীমা	1 0	1 0							1 2	1 2 3	
6	ব্যবসা প্রতিষ্ঠান বীমা	1 0	1 0							1 2	1 2 3	
7	ঋণ বীমা*	1 0	1 0							1 2	1 2 3	
8	গবাদি পশু বীমা	1 0	1 0							1 2	1 2 3	
9	হাঁস মুরগী খামার বীমা	1 0	1 0							1 2	1 2 3	
10	মৎস্য খামার বীমা	1 0	1 0							1 2	1 2 3	
11	গোষ্ঠী বীমা*	1 0	1 0							1 2	1 2 3	

*1) বীমাকারীর মৃত্যু হলে জীবন বীমার সমপরিমান টাকা পাওয়া যায়, 2) দুর্ঘর্টনায় পঙ্গু হলে বীমা প্রতিষ্ঠান বীমার সমপরিমান টাকা প্রদান করে, 4) সম্পদ বীমা বলতে ঘর বাড়ী, বাগান, প্রাইভেটকার ইত্যাদির বীমা করানোকে বোঝানো হয়েছে।
7) বীমা কারীর মৃত্যু হলে ঋণ বীমার মাধ্যমে অপরিশোধিত ঋণের অর্থ মওকুফ হয়ে যায় ইত্যাদি। 11) গোষ্ঠী বীমা বলতে একটি প্রতিষ্ঠানে কর্মরত ব্যক্তিদের সম্মিলিত ভাবে কোন বীমার সদস্য হওয়াকে বুঝানো হয়েছে।**একই বীমা একাধীকবার/ একাধীক সদস্যের থাকলে নিচের ঘর ব্যবহার করুন

1	2	3	4	5	6	7	8	9	10	11	12	13
										1 2	1 2 3	
										1 2	1 2 3	
										1 2	1 2 3	
										1 2	1 2 3	

অন্যান্য উল্লেখ করুন

নিম্নের প্রশ্ন গুলো (503-517) শুধুমাত্র গ্রামীন কল্যানের প্রোগ্রাম এরিয়ার জন্য প্রযোজ্য (*2009 এর প্যানেলের খানার জন্য প্রযোজ্য)

503. আপনি গ্রামীণ কল্যানের স্বাস্থ্য বীমা সম্পর্কে শুনেছেন কি?	☐ হ্যাঁ	☐ না			
504. স্বাস্থ্য কর্মীরা কখনও আপনাকে বা আপনার খানার সদস্যদের কে গ্রামীণ কল্যানের স্বাস্থ্য বীমা সম্পর্কে ধারনা দিয়েছিলেন কি ?	☐ হ্যাঁ	☐ না			
505. নগদ বা গ্রামীণ ব্যাংকে আপনার সঞ্চয় থেকে প্রদানের মাধ্যমে কখনও গ্রামীণ কল্যানের স্বাস্থ্য বীমা কার্ড করেছিলেন কি? (না হলে, 517 নং প্রশ্নে যান)	☐ হ্যাঁ	☐ না			
506. 505 এর উত্তর হ্যাঁ হলে, আপনার পরিবার (খানা) কি বর্তমানে গ্রামীন কল্যাণ স্বাস্থ্য কেন্দ্রের সদস্য? (না হলে, 511 নং প্রশ্নে যান)	☐ হ্যাঁ	☐ না			
507. 506 এর উত্তর হ্যাঁ হলে, সর্বশেষ কত মাস আগে কার্ডটি সংগ্রহ/নবায়ন করেছিলেন? (অর্থাৎ কত মাস আগে আপনার সঞ্চয় থেকে/নগদ প্রদান করে কার্ডটি সংগ্রহ করেছিলেন)	☐☐☐				
508. আপনি কত বছর ধরে গ্রামীন কল্যাণের সদস্য?	☐☐				
509. গ্রামীন কল্যাণের স্বাস্থ্যকর্মীরা কখনো আপনার বাড়িতে এসে আপনার পরিবারের স্বাস্থ্য পরীক্ষা বা সুস্বাস্থ্য সম্পর্কে ধারণা দিয়েছিলেন কি?	☐ হ্যাঁ	☐ না			
510. 509 এর উত্তর যদি হ্যাঁ হয় তবে বিগত তিন মাসে কত বার আপনার বাড়িতে স্বাস্থ্য পরীক্ষা করতে এসেছিলেন?	☐☐				
511. 506 এর উত্তর না হলে কার্ডটির মেয়াদ কত মাস আগে উত্তীর্ণ হয়েছিল?	☐☐				
512. গ্রামীন কল্যানের স্বাস্থ্য কেন্দ্র থেকে স্বাস্থ্য সেবা গ্রহন করেন কি? (না হলে, 516 নং প্রশ্নে যান)	☐ হ্যাঁ	☐ না			
513. গ্রামীণ কল্যানের স্বাস্থ্য কেন্দ্র যে সকল সেবা প্রদান করে, তা আপনার মতে কেমন? (পর্যাপ্ত নয় হলে 514 নং এবং পর্যাপ্ত ও মোটামুটি হলে 515 এ যান)	☐ পর্যাপ্ত	☐ মোটামুটি	☐ পর্যাপ্ত নয়		
514. পর্যাপ্ত না হলে আর কি কি স্বাস্থ্য সেবা দেওয়া দরকার বলে আপনি মনে করেন ?					
515. গ্রামীন কল্যানের স্বাস্থ্য কেন্দ্র থেকে যে সকল সেবা প্রদান করা হয় তার মান আপনার মতে কেমন?	☐ খুবই ভালো	☐ ভালো	☐ মোটামুটি	☐ খারাপ	☐ খুবই খারাপ
516. 512 এর উত্তর না হলে জিজ্ঞাসা করুন কেন গ্রামীন কল্যানের স্বাস্থ্য কেন্দ্র থেকে স্বাস্থ্য সেবা গ্রহণ করেন না? (তিনটি প্রধান কারণ উল্লেখ করুন)(কোড-28)	1 ☐☐	2 ☐☐	3 ☐☐		
517. 505 এর উত্তর না হলে জিজ্ঞাসা করুন গ্রামীণ কল্যানের সদস্য না হওয়ার কারণ কি? (একাধিক উত্তর প্রযোজ্য) (কোড-28)	1 ☐☐	2 ☐☐	3 ☐☐		

518. কোন প্রক্রিয়ায় স্বাস্থ্য কার্ড সংগ্রহ করেছিলেন?

- ☐ গ্রামীণ ব্যাংকের সঞ্চয়ী হিসাব হতে স্বাস্থ্য কার্ড বাবদ টাকা প্রদানের সম্মতি জানিয়ে স্বাক্ষর প্রদানের মাধ্যমে।
- ☐ গ্রামীণ কল্যানের স্বাস্থ্য কেন্দ্রে সেবা গ্রহনের সময়।
- ☐ গ্রামীণ কল্যানের কর্মীরা বাড়ীতে এসে স্বাক্ষর সংগ্রহ করেছিলেন।
- ☐ গ্রামীণ কল্যানের কর্মীদের নগদ প্রদান করে।
- ☐ অন্যান্য (উল্লেখ করুন)

519. কার্ডটি এ বছর নবায়ন করেছেন কি?

☐ হ্যাঁ (পরবর্তী সেকশনে যান) ☐ না

520. আপনি কেন ক্ষুদ্র স্বাস্থ্য বীমার কার্ডটি নবায়ন করেননি? (একাধিক উত্তর প্রযোজ্য)

- ☐ বীমা কার্ড ব্যবহারের কোন সুযোগ পাই না।
- ☐ বীমা কার্ড নবায়নের জন্য যে পরিমান টাকা দরকার তা যোগার করতে না পারা।
- ☐ ক্ষুদ্র স্বাস্থ্য বীমা কেন্দ্র জটিল রোগের চিকিৎসা প্রদান করতে পারে না।
- ☐ যারা কার্ড ক্রয় করে তাদেরকে অবহেলার দৃষ্টিতে দেখে।
- ☐ স্বাস্থ্য সেবার গুনগত মান ভাল নয়।
- ☐ যারা কার্ড ক্রয় করে এবং যারা ক্রয় করে না সকলে একই সুযোগ সুবিধা পায়।
- ☐ রেফারেল সার্ভিস উপকারে আসে না।
- ☐ এম বি বি এস ডাক্তার পাওয়া যায় না।
- ☐ কার্ডের মূল্য বাড়ানো হয়েছে।
- ☐ কার্ডটির এখনও মেয়াদ আছে ।
- ☐ স্বাস্থ্য বাবদ বড় ধরনের ব্যয় হবে এমন প্রত্যাশা করি না
- ☐ একই ধরনের স্বাস্থ্য সেবা সরকারী/প্রাইভেট স্বাস্থ্য কেন্দ্রে পাওয়া যায়।
- ☐ অন্যান্য (উল্লেখ করুন) ঃ

521. নবায়ন না করে থাকেন তবে আর নবায়ন করার ইচ্ছা আছে কি ?

☐ হ্যাঁ (পরবর্তী সেকশনে যান) ☐ না

517. না হলে কেন নবায়ন করার ইচ্ছা নেই ? (একাধিক উত্তর প্রযোজ্য)

- ☐ বীমা কার্ড ব্যবহারের কোন সুযোগ পাই না।
- ☐ বীমা কার্ড নবায়নের জন্য যে পরিমান টাকা দরকার তা যোগার করতে না পারা।
- ☐ ক্ষুদ্র স্বাস্থ্য বীমা কেন্দ্র জটিল রোগের চিকিৎসা প্রদান করতে পারে না।
- ☐ যারা কার্ড ক্রয় করে তাদেরকে অবহেলার দৃষ্টিতে দেখে।
- ☐ স্বাস্থ্য সেবার গুনগত মান ভাল নয়।
- ☐ যারা কার্ড ক্রয় করে এবং যারা ক্রয় করে না সকলে একই সুযোগ সুবিধা পায়।
- ☐ রেফারেল সার্ভিস উপকারে আসে না।
- ☐ এম বি বি এস ডাক্তার পাওয়া যায় না
- ☐ কার্ডের মূল্য বাড়ানো হয়েছে।
- ☐ অন্যান্য (নির্দিষ্ট করুন)

অন্যান্য উল্লেখ করুন

সেকশন-6 ঃ ঋণ সংক্রান্ত তথ্য **601.** বর্তমানে আপনার খানার কেউ কোন ক্ষুদ্রঋণ (Microcredit) প্রতিষ্ঠানের সদস্য কি? হ্যাঁ 1 না 2 (602 নং প্রশ্নে যান)

1	2	3	4	5	6	7	8		9	10	11	12		13	14		15	16
সদস্য কোড	কোন ক্ষুদ্রঋণ/ এনজিও প্রতিষ্ঠানের সদস্য? (কোড-29)	কত বছর ধরে সদস্য?	এ যাবৎকাল পর্যন্ত সর্বমোট কতবার ঋণ নিয়েছেন? (না নিয়ে থাকলে 0 লিখুন এবং 15 নং প্রশ্নে যান)	সর্বশেষ মোট কত টাকা ঋণ হিসেবে গ্রহন করেছেন? (টাকা)	সপ্তাহে কত টাকা কিস্তি দিতে হয়?	মোট কয়টি কিস্তিতে পুরো ঋণ পরিশোধীত হবে? (সংখ্যা)	কিস্তির টাকা কিভাবে পরিশোধ করেন? (কোড-30)		এ পর্যন্ত কত কিস্তি পরিশোধ হয়েছে?	সর্বশেষ ঋন এর ক্ষেত্রে কিস্তির টাকা পরিশোধ করতে ব্যর্থ হয়েছেন কি? 1. হ্যাঁ 2. না 3. পরিশোধের সময় হয়নি (উত্তর না হলে 13 নং এ যান)	হ্যাঁ হলে কত কিস্তি?	কেন কিস্তির টাকা পরিশোধ করতে ব্যর্থ হয়েছিলেন? (কোড-31)		বর্তমানে অপরিশোধিত ঋণের পরিমাণ কত? (টাকা)	সর্বশেষ ঋণের টাকা কোথায় বিনিয়োগ করেছিলেন? (কোড-32)		সপ্তাহে সঞ্চয় বাবদ কত টাকা জমা দিতে হয়?	মোট সঞ্চয়ের পরিমান (টাকা)
							উৎস 1	উৎস 2				উৎস 1	উৎস 2		উৎস 1	উৎস 2		
										1 2 3								
										1 2 3								
										1 2 3								
										1 2 3								

602. আপনি কি গত দুই বছরে NGO প্রদত্ত ক্ষুদ্র ঋণ ছাড়া অন্য কোন উৎস থেকে ঋণ গ্রহন করেছেন? হ্যাঁ 1 না 2
যদি হ্যাঁ হয় তবে নিম্নের Column গুলো পূরণ করুন-

1	2	3	4	5	6		7		8	9	
সদস্য কোড	কোন প্রতিষ্ঠান বা ব্যাক্তি থেকে ঋণ নিয়েছেন? (কোড-33)	ঐ প্রতিষ্ঠান বা ব্যাক্তি থেকে সর্বশেষ ঋণ গ্রহনের পরিমাণ	সুদসহ মোট কত টাকা পরিশোধ করতে হয়েছিলো/হবে?	ঋণের টাকা কি পরিমান পরিশোধ করেছেন? 1. সম্পুর্ন 2. আংশিক 3. ঋণ খেলাপি হয়েছে বা সময়মত পরিশোধ করা হয়নি 4. পরিশোধের সময় হয়নি (3 হলে 7 নং প্রশ্নে যান)	ঋণের টাকা কিভাবে পরিশোধ করেছেন? (কোড-30)		কেন টাকা পরিশোধ করতে ব্যর্থ হয়েছেন? (কোড-31)		বর্তমানে অপরিশোধিত ঋণের পরিমাণ কত? (টাকা)	গত দুই বছরে সর্বশেষ ঋণের টাকা কোথায় বিনিয়োগ করেছিলেন? (কোড-32) (প্যানেল এর ক্ষেত্রে 2009 এর আগস্ট এর পর থেকে)	
					উৎস 1	উৎস 2	উৎস 1	উৎস 2		উৎস 1	উৎস 2
				1 2 3 4							
				1 2 3 4							
				1 2 3 4							
				1 2 3 4							

অন্যান্য উল্লেখ করুন

সেকশন-7 ঃ খরচ সংক্রান্ত তথ্য

701. খানাতে কত জন সদস্য নিয়মিত খায়? ☐☐ জন

702. খাদ্য ব্যয়

কোড	খাদ্য তালিকা	মোট ভোগের পরিমাণ		একক প্রতি ক্রয় মূল্য	
গত এক সপ্তাহে খাদ্য শস্য		একক 1 = গ্রাম, 2 = কেজি, 3 = মণ, 4 = সংখ্যা	ভোগের পরিমাণ	একক 2 = কেজি/লিটার, 3 = মণ, 4= সংখ্যা	বাজার মূল্য (টাকা)
1	চাল	1 2 3 4	.	2 3 4	.
2	আটা	1 2 3 4	.	2 3 4	.
3	ময়দা	1 2 3 4	.	2 3 4	.
4	চিড়া/মুড়ি	1 2 3 4	.	2 3 4	.
5	সুজি	1 2 3 4	.	2 3 4	.
গত এক সপ্তাহে ডাল জাতীয় শস্য					
6	মশুর ডাল	1 2 3 4	.	2 3 4	.
7	ছোলার ডাল	1 2 3 4	.	2 3 4	.
8	মাস কলাই	1 2 3 4	.	2 3 4	.
9	খেসারী	1 2 3 4	.	2 3 4	.
10	মুগ	1 2 3 4	.	2 3 4	.
11	বুট	1 2 3 4	.	2 3 4	.
12	এ্যাংকর	1 2 3 4	.	2 3 4	.
গত এক সপ্তাহে ভোজ্য তৈল					
13	সয়াবিন	1 2 3 4	.	2 3 4	.
14	সরিষা	1 2 3 4	.	2 3 4	.
15	ডালডা	1 2 3 4	.	2 3 4	.
16	ঘি	1 2 3 4	.	2 3 4	.
17	পাম ওয়েল	1 2 3 4	.	2 3 4	.
18	তিল এর তেল	1 2 3 4	.	2 3 4	.
19	অন্যান্য	1 2 3 4	.	2 3 4	.

কোড	খাদ্য তালিকা	মোট ভোগের পরিমাণ		একক প্রতি ক্রয় মূল্য	
গত এক মাসে মাংস, ডিম ও দুধ		একক 1 = গ্রাম, 2 = কেজি, 3 = মণ, 4 = সংখ্যা	ভোগের পরিমাণ	একক 2 = কেজি/লিটার, 3 = মণ, 4= সংখ্যা	বাজার মূল্য (টাকা)
20	গরুর মাংস	1 2 3 4	.	2 3 4	.
21	মহিষের মাংস	1 2 3 4	.	2 3 4	.
22	মুরগীর মাংস (দেশী)	1 2 3 4	.	2 3 4	.
23	মুরগীর মাংস (ফার্ম)	1 2 3 4	.	2 3 4	.
24	হাঁসের মাংস	1 2 3 4	.	2 3 4	.
25	খাসীর মাংস	1 2 3 4	.	2 3 4	.
26	কবুতরের মাংস	1 2 3 4	.	2 3 4	.
27	ডিম	1 2 3 4	.	2 3 4	.
28	দুধ	1 2 3 4	.	2 3 4	.
29	কলিজা/মগজ	1 2 3 4	.	2 3 4	.
গত এক মাসে অন্যান্য খাবার					
30	চিনি/গুড়	1 2 3 4	.	2 3 4	.
31	গুঁড়ো দুধ	1 2 3 4	.	2 3 4	.
32	লবন	1 2 3 4	.	2 3 4	.
33	হলুদ	1 2 3 4	.	2 3 4	.
34	মরিচ (কাঁচা)	1 2 3 4	.	2 3 4	.
35	মরিচ (শুকনা)	1 2 3 4	.	2 3 4	.
36	পেয়াজ	1 2 3 4	.	2 3 4	.
37	রসুন	1 2 3 4	.	2 3 4	.
38	আলু	1 2 3 4	.	2 3 4	.
39	অন্যান্য সবজি	1 2 3 4	.	2 3 4	.

অন্যান্য উল্লেখ করুন

কোড	খাদ্য তালিকা	মোট ভোগের পরিমাণ		একক প্রতি ক্রয় মূল্য	
গত এক মাসে অন্যান্য খাবার		একক 1 = গ্রাম, 2 = কেজি, 3 = মণ, 4 = সংখ্যা	ভোগের পরিমাণ	একক 2 = কেজি/লিটার, 3 = মণ, 4= সংখ্যা	বাজার মূল্য (টাকা)
40	শাক	1 2 3 4	.	2 3 4	.
41	মাছ (বড়)	1 2 3 4	.	2 3 4	.
42	শুটকি মাছ	1 2 3 4	.	2 3 4	.
43	ফল (পাকা কলা)	1 2 3 4	.	2 3 4	.
44	মিষ্টি/বাতাসা/জিলাপী	1 2 3 4	.	2 3 4	.
45	ভুট্টা	1 2 3 4	.	2 3 4	.
46	অন্যান্য (উল্লেখ করুন)	1 2 3 4	.	2 3 4	.
47		1 2 3 4	.	2 3 4	.
48		1 2 3 4	.	2 3 4	.
49		1 2 3 4	.	2 3 4	.
		1 2 3 4	.	2 3 4	.
		1 2 3 4	.	2 3 4	.
		1 2 3 4	.	2 3 4	.

কোড	খাদ্য তালিকা	ক্রয় বাবদ ব্যয় (টাকা)
50	হোটেল/রেস্টুরেন্ট থেকে কেনা খাবার	
51	কোমল পানীয়	
52	বিড়ি/সিগারেট	
53	পান/তামাক	
54	বিস্কুট	
55	চানাচুর	
56	অন্যান্য ফল	
57	মসলা	
58	চা/চা পাতা	
59	মাছ (ছোট)	
60	অন্যান্য (উল্লেখ করুন)	

অন্যান্য উল্লেখ করুন

সেকশন 703. খাদ্য বহির্ভূত ব্যয় (গত 12 মাসের হিসাব)

1	2	3	4	5
কোড	ব্যয়ের খাত	সাপ্তাহিক (টাকা)	মাসিক (টাকা)	বাৎসরিক (টাকা)
1	পোশাক-পরিচ্ছদ বাবদ নিয়মিত ব্যয়			
2	প্রসাধনী (সাবান/সোডা/পাউডার/কসমেটিক/টুথপেষ্ট/মাজন/ নারিকেল তেল ইত্যাদি)			
3	হাঁড়ি-পাতিল, থালা-বাসন ইত্যাদি			
4	লেপ/তোষক/বালিশ/চাদর/বিছানা			
5	আসবাবপত্র ও সরঞ্জামাদি			
6	হেরিকেন/লণ্ঠন/কুপি/টর্চ/বাল্ব ইত্যাদি ক্রয়			
7	কেরোসিন/ম্যাচ/মোমবাতি ইত্যাদি			
8	বিদ্যুত খরচ			
9	জেনারেটরের বিল			
10	জ্বালানী কাঠ			
11	গ্যাস বিল			
12	যাতায়াত (নিয়মিত)			
13	খানায় ব্যবহৃত সামগ্রীর মেরামত			
14	ঘর মেরামত ও রক্ষণাবেক্ষণ			
15	চিকিৎসা বাবদ ব্যয়			
16	ঘর-বাড়ির উন্নয়ন ও বর্দ্ধিতকরণ			
17	সৌর বিদ্যুতের স্থাপনা/কিস্তি			
18	জমি/বাড়ি ক্রয়			
19	জমি ও চৌকিদারী খাজনা			

1	2	3	4	5
কোড	ব্যয়ের খাত	সাপ্তাহিক (টাকা)	মাসিক (টাকা)	বাৎসরিক (টাকা)
20	ক্ষুদ্রঋণের কিস্তি			
21	চাঁদা/দান			
22	বিনোদন/ব্যক্তিগত ভ্রমণ (পোশাক-পরিচ্ছদ সহ)			
23	শিক্ষা ও পেশাগত সেবা (স্কুল-কলেজের বেতন)			
24	খাতা/কলম/বই/মনিহারী দ্রব্য			
25	মোবাইল/টেলিফোন (বিল)			
26	বিবাহ/জন্ম/মৃত্যুজনিত অনুষ্ঠানাদি (পোশাক-পরিচ্ছদ সহ)			
27	সামাজিক/ধর্মীয় অনুষ্ঠানাদি (পোশাক-পরিচ্ছদ সহ)			
28	যানবাহনের জ্বালানী/মেরামত বাবদ ব্যয়			
29	বাড়ি ভাড়া			
30	কর্মচারীর বেতন			
31	ডিস বিল/কেবল সংযোগের বিল			
32	গৃহপরিচারক/ গৃহপরিচারিকার বেতন			
33	উপহার সামগ্রী			
34	যাকাত/ফিতরা			
35	সেলুন/পার্লার			
36	লন্ড্রি			
37	অন্যান্য ব্যয় (উল্লেখ করুন)			

704. গত ১২ মাসে আপনার খানাতে খাদ্য দ্রব্য ও অন্যান্য খরচে কোন বড় ধরনের পরিবর্তন হয়েছিল কি? হ্যাঁ 1 না 2

705. হ্যাঁ হলে কেন (প্রধান কারণ উল্যেখ করুন)

অন্যান্য উল্লেখ করুন

সেকশন 8: বিভিন্ন উৎস থেকে আয় 801. উৎপাদিত ফসলের পরিমাণ (গত ১২ মাসে)

1	2	3	4	5-6		7-8		9	10	11-13			14
ফসল কোড	ফসলের বিবরণ	মোট জমির পরিমান যে পরিমান জমিতে এই ফসল চাষ করা হয়েছে পরিমান (শতাংশ)	যে পরিমান জমিতে এই ফসল চাষ হয়েছে তাতে নিজ অংশ কতটুকু? (শতাংশ)(মালিকানাধীন)	উৎপাদনের পরিমান (বিভিন্ন ধরনের জমি থেকে প্রাপ্ত ফসলের নিজের অংশ উলেখ করুন)		ফসলের মূল্য		মজুরী বাবদ দেয় ফসল (পরিমান)	সেচ বাবদ দেয় ফসল (পরিমান)	বিক্রয়কৃত ফসল			উপজাত থেকে আয়
				2= কেজি 3= মণ 4= সংখ্যা/পন/কাঁদি	পরিমাণ	2= কেজি 3= মণ 4= সংখ্যা/পন/কাঁদি	একক প্রতি মূল্য (টাকা)			2= কেজি 3= মণ 4= সংখ্যা/পন/কাঁদি	পরিমান	প্রাপ্ত অর্থ	
1	আউশ			2 3 4		2 3 4				2 3 4			
2	আমন			2 3 4		2 3 4				2 3 4			
3	বোরো			2 3 4		2 3 4				2 3 4			
4	সুবাসিত ধান			2 3 4		2 3 4				2 3 4			
5	গম			2 3 4		2 3 4				2 3 4			
6	ভুট্টা			2 3 4		2 3 4				2 3 4			
7	পেয়াজ			2 3 4		2 3 4				2 3 4			
8	মরিচ			2 3 4		2 3 4				2 3 4			
9	রসুন			2 3 4		2 3 4				2 3 4			
10	আদা			2 3 4		2 3 4				2 3 4			
11	অন্যান্য মসলা			2 3 4		2 3 4				2 3 4			
12	কলা			2 3 4		2 3 4				2 3 4			
13	আনারস			2 3 4		2 3 4				2 3 4			
14	কাঁঠাল/আম			2 3 4		2 3 4				2 3 4			
15	আখ			2 3 4		2 3 4				2 3 4			
16	আলু			2 3 4		2 3 4				2 3 4			
17	শাক-সবজি			2 3 4		2 3 4				2 3 4			
18	পাট			2 3 4		2 3 4				2 3 4			
19	তামাক			2 3 4		2 3 4				2 3 4			
20	সুপারী/পান			2 3 4		2 3 4				2 3 4			
21	ডাল			2 3 4		2 3 4				2 3 4			
22	তৈলবীজ			2 3 4		2 3 4				2 3 4			
23	অন্যান্য (উল্লেখ করুন)			2 3 4		2 3 4				2 3 4			

অন্যান্য উল্লেখ করুন

802. উৎপাদিত ফসল বাবদ ব্যয় (গত ১২ মাসে)

1	2	3	4	5	6	7	8	9	10
ফসল কোড	ফসলের বিবরণ	সার বাবদ ব্যয় (টাকা)	সেচ/পানি বাবদ ব্যয় (টাকা)	বীজ বাবদ ব্যয় (টাকা)	কীটনাশক বাবদ ব্যয় (টাকা)	শ্রমিক বাবদ ব্যয় (টাকা)	লাঙ্গল খরচ (টাকা)	পাওয়ার টিলার/থ্রেসা ভাড়া (টাকা)	অন্যান্য আনুসঙ্গিক ব্যয় (টাকা)
1	আউশ								
2	আমন								
3	বোরো								
4	সুবাসিত ধান								
5	গম								
6	ভুট্টা								
7	পেয়াজ								
8	মরিচ								
9	রসুন								
10	আদা								
11	অন্যান্য মসলা								
12	কলা								
13	আনারস								
14	কাঁঠাল/আম								
15	আখ								
16	আলু								
17	শাক-সবজি								
18	পাট								
19	তামাক								
20	সুপারী/পান								
21	ডাল								
22	তৈলবীজ								
23	অন্যান্য (উল্লেখ করুন)								

অন্যান্য উল্লেখ করুন

গবাদি পশু ঃ সম্পদ উৎপাদন ও আয় (803. পশু সম্পদের বিবরণঃ)

1	2	3	4	5-8				9	10-12			13-15			16-17	
				গত ১২ মাসে ক্রয়				গত 12 মাসে ভোগ	গত ১২ মাসে বিক্রয়			গত ১২ মাসে পশু উৎপাদন খরচ			গত ১২ মাসে পশু মৃত্যু বা হারানোজনিত ক্ষতি	
কোড	বিবরণ	বর্তমান সংখ্যা	মোট বাজার মূল্য	সংখ্যা	মোট ব্যয়িত অর্থ (টাকা)	অর্থের উৎস (কোড-34)	ক্রয়ের কারণ (কোড-35)	(বাজার মূল্য)	সংখ্যা	মোট প্রাপ্ত অর্থ (টাকা)	বিক্রয়ের কারণ (কোড-36)	খাদ্য ক্রয়	চিকিৎসা (ইনজেকশন সহ)	অন্যান্য খরচ	সংখ্যা	(টাকা)
1	গরু															
2	মহিষ															
3	ভেড়া/ছাগল															
4	হাঁস															
5	মুরগী															
6	কবুতর															
7	অন্যান্য (উল্লেখ করুন)															

804. বনজ সম্পদ ও অন্যান্য কৃষি উৎপাদন থেকে আয় (ভোগসহ) ঃ গত 12 মাসে

1	2	3	4	5	6	7	8
কোড	বিবরণ	বর্তমান পরিমাণ	মোট বাজার মূল্য (টাকা)*	গত 12 মাসে বিক্রয় (পরিমাণ)	গত 12 মাসে বিক্রয় বাবদ প্রাপ্ত অর্থ (টাকা)	গত 12 মাসে ব্যবহার (ভোগ) টাকা	গত 12 মাসে উৎপাদন খরচ
1	গাছ (সংখ্যা)						
2	মাছ চাষ (কেজি)						
3	ফল (কেজি)						
4	ডিম (সংখ্যা)						
5	দুধ (কেজি)						
6	বিক্রয়যোগ্য বাঁশ						

805A. দিন মজুরী/কৃষি মজুরী থেকে আয় (গত 12 মাসে)

1	2	3	4
সদস্য কোড	গত এক বছরে কত মাস কাজ করেছেন	কর্ম মাসে গড়ে কত টাকা আয় করেছেন	গত এক বছরে আয়

অন্যান্য উল্লেখ করুন

805B. অন্যান্য উৎস থেকে আয় (গত 12 মাসে)

1	2	3	4
কোড	বিবরণ	গত এক বছরে কত মাস কাজ করেছেন	গত 12 মাসে আয় (টাকা)
1	ব্যবসা থেকে আয়		
2	দোকান থেকে আয়		
3	রিক্সা/ভ্যান/নৌকা/মটর সাইকেল/অটো রিক্সা ইত্যাদি চালানো থেকে আয়		
4	আভ্যন্তরীন রেমিটেন্স		
5	বৈদেশিক রেমিটেন্স		
6	কারখানা থেকে আয়		
7	সুদী কারবার থেকে আয়		
8	গার্মেন্টস থেকে আয়		
9	অন্যান্য (উল্লেখ করুন)		

806. বিভিন্ন ধরনের ভাড়া/বন্ধকী থেকে আয় ও ব্যয়ের হিসাব ঃ (গত 12 মাসে)

1	2	3	4	5
কোড	ভাড়া/বন্ধকী দেওয়া সম্পদ ও যন্ত্রপাতির নাম	পরিমান	ভাড়া/বন্ধকী থেকে গত 12 মাসে মোট প্রাপ্তি (টাকা)	ভাড়া/বন্ধকী দেওয়া সম্পদের মেরামত ও অন্যান্য খরচ বাবদ12 মাসে ব্যয় (টাকা)
1	জমি বন্ধকী//ইজারা/লিজ (শতাংশ)			
2	বাড়ী ভাড়া (শতাংশ)			
3	দোকান/গুদাম ভাড়া (শতাংশ)			
4	সেচ যন্ত্র ভাড়া (সংখ্যা)			
5	পাওয়ার টিলার /ট্রাক্টর ভাড়া (সংখ্যা)			
6	হালের বলদ ভাড়া (সংখ্যা)			
7	রিক্সা/জাল/নৌকা/মটরসাইকেল/অটোরিক্সা ভাড়া			
8	স্বর্ণালংকার বন্ধকী (ভরি)			
9	অন্যান্য (উল্লেখ করুন)			

807. চাকুরী, পেনশন এবং সামাজিক নিরাপত্তা কর্মসূচী থেকে আয়

1	2	3
কোড	চাকুরী/কর্মসূচী/সামাজিক নিরাপত্তার ধরন	গত এক বছরে মোট প্রাপ্তি
1	দান/সহায়তা (যাকাত, ফিতরা)	
2	কাজের বিনিময়ে খাদ্য কর্মসূচী	
3	বয়স্ক ভাতা	
4	বিধবা ভাতা	
5	বৃত্তি/উপবৃত্তি	
6	চাকুরী	
7	পেনশন	
8	কাবিটা/৪০ দিন কর্মসূচী/ভিজিডি/ভিজিএফ/জিআর/টিআর	
9	প্রতিবন্ধী /মুক্তিযোদ্ধা ভাতা	
10	অন্যান্য পেশাজীবি আয় (উল্লেখ করুন)	

808. গত 12 মাসে খাদ্যদ্রব্য ভোগের ক্ষেত্রে আপনার খানার জন্য নিচের কোন টি প্রযোজ্য?

- ☐ সারা বছর খাদ্য ঘাটতি ছিল
- ☐ মাঝে মাঝে খাদ্য ঘাটতি ছিল
- ☐ ঘাটতিও ছিল না আবার অতিরিক্তও ছিল না
- ☐ অতিরিক্ত ছিল।

809. ভোগের পরিমান

1. বর্তমানে কয়বেলা খান?	2. গত,সেপ্টেম্বর থেকে নভেম্বর (আশ্বিন/কার্তিক) সময় কয়বেলা খেয়েছেন?
☐ মাঝে মাঝে না খাওয়া	☐ মাঝে মাঝে না খাওয়া
☐ দুইবেলা আধপেট খাওয়া	☐ দুইবেলা আধপেট খাওয়া
☐ শুধুই দুইবেলা খাওয়া	☐ শুধুই দুইবেলা খাওয়া
☐ তিনবেলা আধপেট খাওয়া	☐ তিনবেলা আধপেট খাওয়া
☐ তিনবেলা ভরপেট খাওয়া	☐ তিনবেলা ভরপেট খাওয়া

অন্যান্য উল্লেখ করুন

সেকশন 9. পরিবারের বর্তমান সম্পদের বিবরণ

901. পরিবারের জমি

1	2	3	4	5-6		7-8-9			10-11		12-13		14	15	16	17
কোড	জমির বর্ণনা	বর্তমানে মালিকানাধীন জমির পরিমাণ (শতাংশ)	বর্তমান বাজারমূল্য	গত 12 মাসে ক্রয়		গত 12 মাসে বিক্রয়			বর্তমানে বন্ধকীতে দেয়া জমির পরিমাণ		বর্তমানে বন্ধকীতে নেয়া জমির পরিমাণ (শতাংশ)		বর্তমানে বর্গায় দেয়া জমির পরিমাণ (শতাংশ)	বর্তমানে বর্গায় নেওয়া জমির পরিমাণ (শতাংশ)	বর্তমানে লিজ/ভাড়ায় দেয়া জমির পরিমাণ (শতাংশ)	বর্তমানে লিজ/ভাড়ায় নেওয়া জমির পরিমাণ (শতাংশ)
				পরিমাণ (শতাংশ)	ক্রয় বাবদ ব্যয়িত অর্থ (টাকায়)	পরিমাণ (শতাংশ)	বিক্রয় বাবদ প্রাপ্ত অর্থ (টাকায়)	বিক্রয়ের প্রধান কারণ (কোড-36)	পরিমাণ (শতাংশ)	প্রাপ্ত অর্থ (টাকায়)	পরিমাণ (শতাংশ)	দেয় অর্থ (টাকা)				
1	বসতভিটা															
2	চাষের অধীন/আবাদযোগ্য															
3	পতিত															
4	বাঁশঝাড়															
5	সুপারী/ফলের বাগান															
6	মাছ চাষাধীন জলাশয়															
7	অন্যান্য জলাশয়															
8	অন্যান্য (উল্লেখ করুন)															
9	মোট জমির পরিমান															

902. কৃষি যন্ত্রপাতি

1	2	3	4	5	6	7
কোড	বিবরণ	বর্তমানে কয়টি আছে (সংখ্যা)	বর্তমান বাজার মূল্য (টাকা)	গত 12 মাসে ক্রয় (টাকা)	গত 12 মাসে বিক্রয় (টাকা)	বিক্রয়ের প্রধান কারণ (কোড-36)
1	সেচ যন্ত্র(**সব ধরনের সেচ যন্ত্র**)					
2	পাওয়ার টিলার/ট্রাক্টর					
3	ধান মাড়াই যন্ত্র					
4	আখ মাড়াই কল					
5	লাঙ্গল					
6	জাল					
7	অন্যান্য (উল্লেখ করুন)ঃ					

অন্যান্য উল্লেখ করুন

903. যানবাহন

1	2	3	4	5	6	7
কোড	বিবরণ	বর্তমান সংখ্যা	বর্তমান বাজার মূল্য (টাকা)	গত 12 মাসে ক্রয় (টাকা)	গত 12 মাসে বিক্রয় (টাকা)	বিক্রয়ের প্রধান কারণ (কোড-36)
1	গরু/মহিষের গাড়ী					
2	নৌকা/যন্ত্রচালিত নৌকা					
3	রিক্সা/ভ্যান					
4	বাইসাইকেল					
5	মোটরসাইকেল					
6	বেবী ট্যাক্সি/অটোরিক্সা					
7	বাস/ট্রাক					
8	নছিমন/করিমন					
9	প্রাইভেট কার					
10	অন্যান্য (উল্লেখ করুন)					

905. দোকান/ব্যবসায় নিয়োজিত সম্পদ

*নোট : 901 এ অন্তর্ভুক্ত হয়নি এমন জমি।

1	2	3	4	5	6	7
1	জমি*					
2	দোকান ঘর					
3	গুদাম ঘর					
4	দোকান/গুদামে রাখা বিক্রয়যোগ্য পণ্য					
5	আসবাবপত্র/যন্ত্রপাতি					
6	কম্পিউটার					
7	অন্যান্য (উল্লেখ করুন)					

906. আর্থিক সম্পদ

1	2	3	4
কোড	বিবরণ	(টাকা)	(বলতে অপারগ হলে টিক দিন)
1	ক্ষুদ্রঋণ প্রতিষ্ঠানে জমার পরিমাণ		☐
2	ডি পি এস		☐
3	ব্যাংকে জমার পরিমাণ		☐
4	এফ ডি আর/সঞ্চয় পত্র		☐
5	শেয়ার ডিবেঞ্চার/ প্রাইজ বন্ড		☐
6	ধার দেওয়া অর্থের পরিমাণ		☐
7	অপরের নিকট জমা		☐
8	নগদ অর্থ		☐

904. শিল্পে ব্যবহৃত স্থায়ী সম্পদ

*নোট : 901 এ অন্তর্ভুক্ত হয়নি এমন জমি।

1	2	3	4	5	6	7
কোড	বিবরণ	সংখ্যা	বর্তমান বাজার মূল্য (টাকা)	গত 12 মাসে ক্রয় (টাকা)	গত 12 মাসে বিক্রয় (টাকা)	বিক্রয়ের প্রধান কারণ (কোড-36)
1	জমি* ও ঘর (শতাংশ)					
2	যন্ত্রপাতি					
3	যানবাহন					
4	কম্পিউটার					
5	অন্যান্য (উল্লেখ করুন)					

907. বিবিধ সম্পদ

1	2	3	4	5	6	7
কোড	বিবরণ	বর্তমান সংখ্যা	বর্তমান বাজার মূল্য (টাকা)	গত 12 মাসে ক্রয় (টাকা)	গত 12 মাসে বিক্রয় (টাকা)	বিক্রয়ের প্রধান কারণ (কোড-36)
1	গহনা-স্বর্ণ (কত ভরি)	.				
2	গহনা-রূপা (কত ভরি)	.				
3	ইলেকট্রনিক্স সামগ্রী	.				
4	ফোন/মোবাইল ফোন	.				
5	ফ্রিজ	.				
6	কম্পিউটার	.				
7	আসবাবপত্র	.				
8	গৃহস্থালীর হাঁড়ি-পাতিল	.				
9	সেলাই মেশিন	.				
10	নিজস্ব টিউবওয়েল	.				
11	অন্যান্য (উল্লেখ করুন)	.				

তথ্য সংগ্রহ কারীর নাম: আপনাকে অনেক ধন্যবাদ। স্বাক্ষর

সুপার ভাইজার এর নাম: স্বাক্ষর

অন্যান্য উল্লেখ করুন
